# Age-Dependent Auditory Processing Deficits after Cochlear Synaptopathy Depend on Auditory Nerve Latency and the Ability of the Brain to Recruit LTP/BDNF

**DOI:** 10.3390/brainsci10100710

**Published:** 2020-10-06

**Authors:** Philine Marchetta, Daria Savitska, Angelika Kübler, Giulia Asola, Marie Manthey, Dorit Möhrle, Thomas Schimmang, Lukas Rüttiger, Marlies Knipper, Wibke Singer

**Affiliations:** 1 Department of Otolaryngology, Head & Neck Surgery, Tübingen Hearing Research Centre (THRC), Molecular Physiology of Hearing, University of Tübingen, Elfriede-Aulhorn-Straße 5, 72076 Tübingen, Germany; philine.marchetta@uni-tuebingen.de (P.M.); daria.savitska@uni-tuebingen.de (D.S.); kuebler.angelika@gmail.com (A.K.); guiliaasola@hotmail.it (G.A.); marie.manthey@tufts.edu (M.M.); dorit.moehrle@googlemail.com (D.M.); lukas.ruettiger@uni-tuebingen.de (L.R.); wibke.singer@uni-tuebingen.de (W.S.); 2 Instituto de Biologíay Genética Molecular, Universidad de Valladolid y Consejo Superior de Investigaciones Científicas, E-47003 Valladolid, Spain; schimman@ibgm.uva.es

**Keywords:** central compensation, cochlear synaptopathy, auditory nerve latency, age-related hearing loss, activity dependent BDNF, long term potentiation

## Abstract

Age-related decoupling of auditory nerve fibers from hair cells (cochlear synaptopathy) has been linked to temporal processing deficits and impaired speech recognition performance. The link between both is elusive. We have previously demonstrated that cochlear synaptopathy, if centrally compensated through enhanced input/output function (neural gain), can prevent age-dependent temporal discrimination loss. It was also found that central neural gain after acoustic trauma was linked to hippocampal long-term potentiation (LTP) and upregulation of brain-derived neurotrophic factor (BDNF). Using middle-aged and old BDNF-live-exon-visualization (BLEV) reporter mice we analyzed the specific recruitment of LTP and the activity-dependent usage of *Bdnf* exon-IV and -VI promoters relative to cochlear synaptopathy and central (temporal) processing. For both groups, specimens with higher or lower ability to centrally compensate diminished auditory nerve activity were found. Strikingly, *low compensating* mouse groups differed from *high compensators* by prolonged auditory nerve latency. Moreover, low compensators exhibited attenuated responses to amplitude-modulated tones, and a reduction of hippocampal LTP and *Bdnf* transcript levels in comparison to high compensators. These results suggest that latency of auditory nerve processing, recruitment of hippocampal LTP, and *Bdnf* transcription, are key factors for age-dependent auditory processing deficits, rather than cochlear synaptopathy or aging per se.

## 1. Introduction

Aging people often experience difficulties in perceiving speech in a noisy environment, even without elevated audiometric thresholds [1]. The development of poor supra-threshold speech processing during aging in humans was attributed to progressive cochlear synaptopathy, similarly to phenomena observed in aging animals [2,3]. In rodents [4,5,6] and humans [7] it was recently shown that loss of afferent auditory fibers (cochlear synaptopathy) can progress over aging or following ‘non-traumatic’ loud sound. This can coincide with hearing deficits even when audiometric thresholds are normal and independently from the loss of outer hair cells. In humans, older people who have maintained good hearing sensitivity, nevertheless frequently report difficulties in acoustically complex environments. This general profile of auditory deficiency, which can occur in individuals with a normal audiogram, is supposed to reflect impaired processing of acoustic temporal cues due to cochlear synaptopathy [8,9]. It was hypothesized that a reduction in the viable population of auditory nerve fibers over age [5,10], particularly of the subpopulation of fibers with a low spontaneous firing rate (low-SR) and a high threshold, might lead to auditory deficits at supra-threshold levels and in challenging listening situations such as in the presence of background noise [2,5,6,11,12,13]. In accord, these fibers with a low SR and a high threshold, that account for 40% of the total amount of fibers, have a high vulnerability to noise and aging [10,14,15,16]. Unlike fibers with a high spontaneous firing rate (high-SR) and a low threshold, low-SR fibers do not contribute to auditory threshold sensitivity [4,5,10]. Therefore, the loss of low-SR fibers would provide a rationale for the numerous studies that report listening deficits in humans despite normal hearing thresholds, e.g., [5,10,17,18,19].

We have previously observed that temporal processing loss was not necessarily attenuated as a consequence of cochlear deafferentation, if reduced auditory input was centrally compensated [6]. In this study [6], stimulus-driven auditory steady state responses (ASSR) were determined, which reflect attention-driven responses in the central auditory pathway to amplitude-modulated tones [20,21,22]. The results demonstrated that only animals with a reduced neural gain over age showed a loss of temporal resolution in auditory processing [6], suggesting neural gain and not cochlear synaptopathy per se as a key factor for temporal processing. This notion is supported by a recent study on the detection abilities for temporally and spectrally modulated stimuli in aging listeners. This study could not confirm a causal link between compromised supra-threshold auditory nerve amplitudes and reduced temporal processing [23].

We also observed that central auditory compensation after acoustic trauma-induced cochlear deafferentation was linked to increased hippocampal long-term potentiation (LTP), and recruitment of activity-dependent *Bdnf* exon-IV and -VI transcription [24], providing a mechanistic explanation for the idea that temporal auditory processing abilities are related to each individual’s cognitive skills [25].

Here we asked how, during aging, cochlear synaptopathy is linked to a central compensation via neural gain, hippocampal LTP and BDNF recruitment.

BDNF-live-exon-visualization (BLEV) reporter mice [26] were used to determine the activity-dependent usage of *Bdnf* exon-IV and -VI promoters through bi-cistronic coexpression of cyan- and yellow-fluorescent-protein (CFP/YFP), respectively [24,26]. Importantly, BLEV mice allow the visualization of *Bdnf* exon-IV-CFP and *Bdnf* exon-VI-YFP transcripts at their sites of translation [26].

We compared young (2.9–6.6 months old), middle-aged (9.4–14.3 months old), and old (15.3–22.5 months old) BLEV mice to uncover differences in central responsiveness at defined states of age-related cochlear synaptopathy. We measured auditory brainstem responses (ABR) to determine wave amplitudes corresponding to the auditory nerve (wave I) and inferior colliculus (IC, wave IV) [27], and examined ASSR, offering an insight in temporal resolution of auditory processing [6]. In addition, we have determined hippocampal LTP and *Bdnf* transcripts to find a correlation between the central auditory adjustment and a memory-dependent facilitation process [24,26]. Strikingly, we found that independently of age and reduced amplitude of the ABR wave I animals with a lower central auditory compensation capacity exhibited a prolonged auditory nerve latency and reduced sensitivity to follow amplitude-modulated tones. These low-compensating groups exhibited reduced LTP after stimulation of the hippocampal Schaffer’s collaterals and attenuated *Bdnf* exon-IV and -VI expression in and around hippocampal capillaries, compared to animals with a higher central capacity to compensate. Prevailed fast auditory nerve processing and the ability of the brain to recruit a LTP/BDNF-dependent facilitation path may therefore play a key role for preventing age-related hearing difficulties caused by cochlear synaptopathy.

## 2. Materials and Methods

### 2.1. Animals

Animal care, procedure, and experimental protocols correspond to national and institutional guidelines and were reviewed and approved by University of Tübingen, Veterinary Care Unit, and the Animal Care and Ethics Committee of the regional board of the Federal State Government of Baden-Württemberg, Germany. All experiments were performed according to the European Union Directive 2010/63/EU for the protection of animals used for experimental and other scientific purposes. In-house bred mice were kept according to the national guidelines for animal care in a specific pathogen free animal facility at 25 °C on a 12 h/12 h light/dark cycle with average noise levels of around 50–60 dB SPL_RMS_.

Female and male homozygous BLEV mice were used and categorized into different age groups. Young animals were between 2.9 and 6.6 months, middle-aged between 9.4 and 14.3 months, and old between 15.3 and 22.5 months old.

The mouse model was generated as described [26]. Briefly, the *Bdnf* exon-IV and -VI sequences, both including the corresponding promoter sequences, were extended by CFP or YFP, respectively, both containing a stop codon. A HA-tag was added to *Bdnf* exon-IV-CFP and a cMyc-tag to *Bdnf* exon-VI-YFP. The translation of the protein-coding *Bdnf* exon-IX was enabled by an internal ribosomal entry site sequence, which keeps the mRNA at the ribosome, despite the presence of a stop codon. Additionally, the growth-associated protein 43, was added to anchor the fluorescent proteins at the site of translation. This allows differential monitoring of the noncoding *Bdnf* exon-IV and *Bdnf* exon-VI by the fluorescent proteins CFP and YFP without interfering with *Bdnf* exon-IX.

### 2.2. Hearing Measurements

Hearing measurements were done under anesthesia 75 mg/kg ketamine hydrochloride (Ketavet^®^, Zoetis GmbH, Berlin, Germany), 5 mg/kg xylazine hydrochloride (Rompun^®^, Bayer Vital GmbH, Leverkusen, Germany), and 0.2 mg/kg atropine (Atropinsulfat B.Braun, Melsungen, Germany) in a soundproof chamber (IAC, Niederkrüchten, Germany), as previously described [28].

The ABR, evoked by short duration sound stimuli, represents the summed activity of neurons in distinct anatomical structures along the ascending auditory pathway [29] and was measured by averaging the evoked electrical response recorded via subcutaneous cranial electrodes. In short, ABR thresholds were elicited with click (100 ms), noise-burst (1 ms duration), or pure-tone stimuli (3 ms, including 1 ms cosine squared rise and fall envelope, 2–45.2 kHz).

ASSRs were measured with amplitude modulated sinusoidal stimuli (carrier frequency 11.31 kHz). The stimuli were presented between −10 and 60 dB in 5 dB steps relative to threshold (re thr). Stimuli were amplitude modulated with 100% modulation depth and 512 Hz modulation frequency. A modulation index was calculated for individual animals by building the ratio between the maximal signal and the baseline (defined as the average of all points except the maximum and the neighboring points). Even if measured threshold normalized, the data were not normalized by threshold for analysis.

### 2.3. Field Excitatory Postsynaptic Potential (fEPSP) Recordings in Hippocampal Slices

Extracellular fEPSP recordings were performed according to standard methods as previously described [30,31].

In brief, 400 µm thick coronal brain slices were cut on a vibratome (Leica VT 1000S) in ice-cold dissection buffer (mM): 127 NaCl, 1.9 KCl, 1.2 KH_2_PO_4_, 26 NaHCO_3_, 10 D-glucose, 2 MgSO_4_, and 1.1 CaCl_2_, constantly saturated with 5% CO_2_ and 95% O_2_ (pH 7.4). Slices were incubated in oxygenated artificial cerebrospinal fluid (ACSF, in mM: 127 NaCl, 1.9 KCl, 1.2 KH_2_PO_4_, 26 NaHCO_3_, 10 D-glucose, 1 MgSO_4,_ 2.2 CaCl_2_; pH 7.4) for 1 h at 30 °C and afterwards stored at room temperature. Recordings were performed in a submerged-type recording chamber (Warner Instruments). Stimulation (TM53CCINS, WPI) and recording (ACSF-filled glass pipettes, 2–3 MΩ) electrodes were positioned in the stratum radiatum (SR) to record Schaffer collateral field excitatory postsynaptic potentials (fEPSPs). Signals were amplified with an Axopatch 200B (Molecular Devices), digitized at 5 kHz with an ITC-16 (HEKA) and recorded using WinWCP from the Strathclyde Electrophysiology Suite. Stimuli (100 µs) were delivered through a stimulus isolator (WPI). For each individual slice the strength of the stimulation (typically between 30–125 µA) was chosen to evoke 40–60% of the maximal response, defined by initial fEPSP slope. Only slices that showed stable fiber volley and fEPSP were used for further recording. The same stimulus intensity was applied during baseline recording (0.067 Hz, 20–30 min) and induction of LTP using 100 stimuli during 1 s (100 Hz, 1 s). The baseline was determined by averaging fEPSP initial slopes from the period before the tetanic stimulation (at least 15 min of stable recording). The level of LTP was determined by averaging fEPSP slopes from the period between 50 and 60 min after the high-frequency stimulation. Before the tetanic stimulation, each slice was used to record input–output relationship (IOR, 25–150 µA in 25 µA steps) and paired-pulse facilitation (PPF, 10–20–50–100–200–500 ms interpulse interval at the same stimulation strength as LTP recordings). IOR changes in fEPSP slope and fiber volley amplitude were normalized within each slice (% from the maximal response at the highest stimulus strength was calculated) and averaged values for each group were plotted against the stimulus intensity. For PPF paired-pulse ratio of EPSP2/EPSP1 slope and amplitude at each interstimulus interval were defined per slice and mean values per group were plotted. EPSP1 was calculated as an average of EPSP1s from all interstimulus intervals for each single slice.

Four traces were averaged for each single data point analyzed.

### 2.4. Tissue Preparation

Tissue preparation was carried out as described in detail previously [32]. In brief, for cochlear cross-section immunohistochemistry, cochleae were isolated, fixed by immersion in 2% paraformaldehyde, 125 mM sucrose in 100 mM phosphate buffered saline (pH 7.4) for 2 h and then decalcified for 45 min in RDO rapid decalcifier (Apex Engineering Products Corporation, Aurora, IL, USA). Cochleae were stored in Sucrose-Hank’s solution rotated at 4 °C overnight before they were embedded in Tissue-tek and cryosectioned in slices of 10 µm, and mounted on SuperFrost*/plus microscope slides before storage at −20 °C.

Brains were fixed by immersion for 48 h in 2% paraformaldehyde (exchange of fixative solution after 24 h) and then stored in 0.4% paraformaldehyde until embedded in 4% agarose. Brains were cut in 60 µm slices with a vibratome (Leica VT 1000S) and stored at −20 °C in cryoprotectant (mix 150 g of sucrose in 200 mL 1× phosphate buffer saline and 150 mL ethylene glycol) until used for immunohistochemistry.

### 2.5. Immunohistochemistry

Immunohistochemistry was carried out as described in detail previously [32]. Antibodies against C-terminal-binding protein 2 (CtBP2)/RIBEYE (rabbit, diluted 1:1500; ARP American Research Products, Inc.™, Waltham, MA, USA) or parvalbumin (PV, rabbit, diluted 1:8000; Abcam, Cambridge, UK #ab11427) were used. Primary antibodies were detected using appropriate Cy3 secondary antibodies (1:1500, Jackson Immuno Research Laboratories, West Grove PA, USA #AB_2338006).

All samples were viewed as previously described [33] using an Olympus BX61 microscope (Olympus, Hamburg, Germany) equipped with epifluorescence illumination and analyzed with CellSens Dimension software (OSIS GmbH, Münster, Germany). To increase spatial resolution, slices were imaged over a distance of 15 µm within an image-stack along the *z*-axis (z-stack), followed by 3-dimensional deconvolution using CellSens Dimension’s built-in algorithm.

### 2.6. Data Analyses

#### 2.6.1. Statistics and Numbers

Unless otherwise stated, all data were presented as group mean with standard deviation (SD) or with standard error of the mean (SEM) for *n* animals per experimental group. Data were tested for normal distribution (Shapiro–Wilk Normality Test, α = 0.05). Differences of the means were compared for statistical significance either by ungrouped two-tailed Student’s *t*-test (parametric)/Mann–Whitney U test (nonparametric), 1-way, or 2-way analysis of variance (ANOVA, parametric)/Kruskal–Wallis test (nonparametric) with α = 0.05 and correction for type 1 error after Tukey-test/Bonferroni’s multiple comparisons test (parametric) or two-stage linear step-up procedure of Benjamini, Krieger, and Yekutieli (nonparametric). In figures, significance is indicated by asterisks (* *p* < 0.05, ** *p* < 0.01, *** *p* < 0.001, **** *p* < 0.0001). n.s. denotes nonsignificant results (*p* > 0.05). All statistical information and *n* numbers can be found in Section 3 and in Table 1. Statistical calculations and visualizations were done with GraphPad Prism.

#### 2.6.2. ABR Analysis

For each individual ear, the peak input–output function and the latency (averaged for intensities between 0 and 30 dB re thr) of the noise-ABR measurements were analyzed as previously described [34].

Two peak classes were selected: (1) early peaks (at 1.2–1.8 ms, wave I) interpreted as the sum of the first stimulus-related action potential within the auditory nerve, and (2) delayed peaks (at 4.1–4.9 ms, wave IV), the response from the auditory midbrain.

For further analysis, the *strength* of the ABR growth function was determined. Therefore the mean of the three values within the supra-threshold growth function with the highest wave amplitude was calculated (more detailed described in [26]) with Excel (Microsoft Excel 2016).

Wave VI/I ratio was calculated by dividing for individual animals at all intensities relative to threshold (re thr) the ABR wave VI amplitude by ABR wave I amplitude. For further analyses, the mean between 20 and 80 dB (re thr) was calculated.

To determine the level of central compensation for individual animals, wave IV/I ratio was plotted against wave I strength. The power function (y = a*x^b) was inserted as a regression line for each group. Only animals with wave I strength smaller than 1.9 µV (which is the mean of all ears in all groups) were subdivided along the black regression line (all ears of all groups). Animals below the line were *low compensators*, while animals above were *high compensators*.

#### 2.6.3. fEPSP Recordings in Hippocampal Slices

Data were analyzed and processed using Clampfit 10 (Molecular Devices) and Microsoft Excel. The data presented per experimental group/condition contained (additionally to mean ± SEM) single dots which showed the fEPSP slope values for each individual brain slice. The *n* indicates the number of slices and animals (slices/animals) used in the analysis.

#### 2.6.4. Fluorescence Analysis of Immunohistochemistry

Pictures acquired from brain sections stained for PV, were analyzed using the free Image J software (NIH, Bethesda, MD, USA). For each section, pictures for each single channel (YFP, CFP, PV) were saved and analyzed independently. For the 10× magnified pictures, after conversion to an 8-bit image, background was reduced using the rolling bar algorithm (available as a tool for Image J) with standard parameters in each single channel picture. Afterwards the integrated density of the fluorescence of CFP and YFP within the picture was calculated. For analysis, data were normalized between both groups. For each individual animal two pictures from duplicate immunostainings were taken and finally the results were averaged to include each animal only once in statistics.

For IHC ribbon counting, pictures were taken from all turns of both ears from duplicate immunohistochemical stainings.

#### 2.6.5. Data Availability

The datasets generated and/or analyzed during the current study are available from the corresponding author upon request.

## 3. Results

### 3.1. Auditory Brainstem Response-Evoked Thresholds Are Elevated in Old but Not Middle-Aged Animals

**Figure 1:**
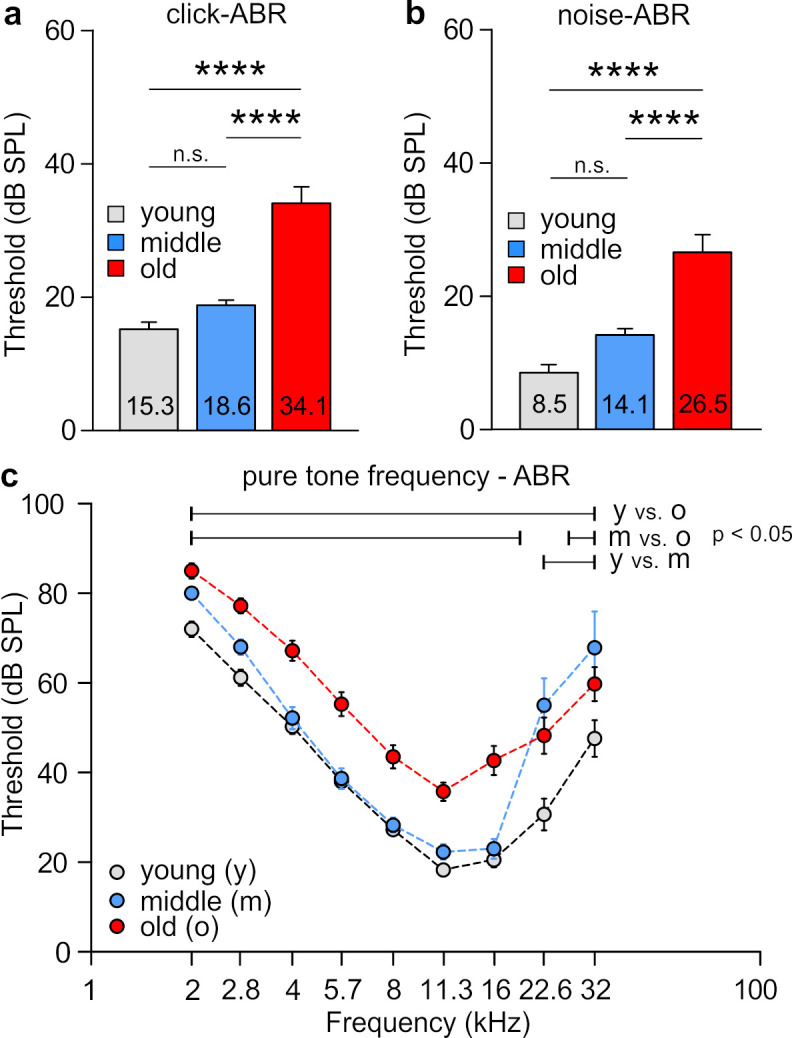
Analysis of the hearing threshold over age. In old animals, hearing thresholds became significantly worse. This was shown for (**a**) click stimuli (1-way ANOVA, F (2, 157) = 39.70, *p* < 0.0001, Tukey’s post hoc test: young vs. middle-aged *p* > 0.05, young vs. old *p* < 0.0001, middle-aged vs. old *p* < 0.0001) and (**b**) noise stimuli (1-way ANOVA, F (2, 157) = 27.23, *p* < 0.0001, Tukey´s post hoc test: young vs. middle-aged *p* > 0.05, young vs. old *p* < 0.0001, middle-aged vs. old *p* < 0.0001). (**c**) With pure-tone frequency-specific auditory stimuli, specifically for high frequencies already middle-aged animals showed increased thresholds compared to young BLEV mice (2-way ANOVA, F (2, 625) = 70.73, *p* < 0.0001, Tukey’s post hoc test: bars indicate significant differences between respective groups in the shown range). *n* for all comparisons: young *n* = 54/27; middle-aged *n* = 28/14; old *n* = 54/27 (ears/animals). **** *p* < 0.0001. Mean ± SEM.

To study the compensation mechanisms underlying age-related synaptopathy, we first compared the hearing thresholds between young (2.9–6.6 months, *n* = 54/27 ears/animals), middle-aged (9.4–14.3 months, *n* = 28/14 ears/animals), and old (15.3–22.5 months, 54/27 ears/animals) BLEV reporter mice. The ABR evoked by low frequency-containing (click), high frequency-containing (noise burst), and pure tone frequency-specific auditory stimuli were tested as described [35]. Old BLEV mice showed a significant increase in threshold compared with young and middle-aged animals measured by click-evoked ABR (Figure 1a; all statistical findings and details of the tests can be found in the figure legends and in Table 1), noise-burst stimuli (Figure 1b), and pure tone frequencies (Figure 1c). Middle-aged BLEV animals showed no different results compared to young BLEV animals upon click and noise stimuli but revealed an increased threshold for frequencies higher than 16 kHz (Figure 1c).

In conclusion, BLEV reporter mice showed an increase of auditory thresholds mainly in the last third of their life span (old animals). This threshold increase started at high frequencies in middle-aged animals but finally all frequencies were affected in old animals.

### 3.2. Late Supra-Threshold ABR Wave Varies in Middle-Aged and Old Animals

**Figure 2:**
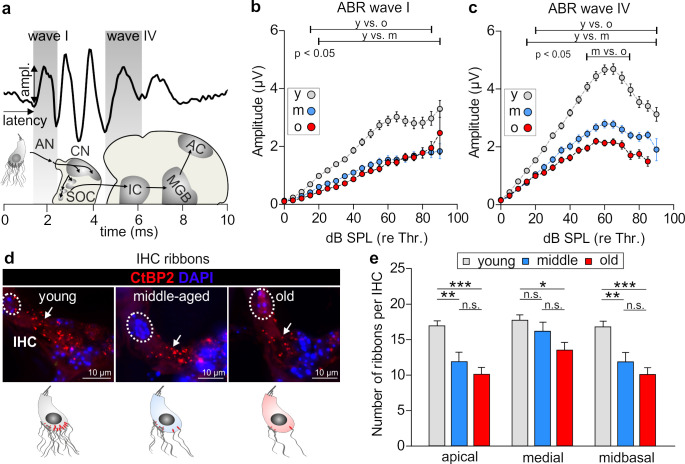
Supra-threshold analyses of auditory brainstem response (ABR) waves I and IV and inner hair cell (IHC) function. (**a**) Schematic drawing of the auditory pathway and correlated stimulus-evoked deflections of ABR waves. AN = auditory nerve, CN = cochlear nucleus, SOC = superior olivary complex, IC = inferior colliculus, MGB = medial geniculate body, AC = auditory cortex. (**b**) ABR wave I amplitude was significantly reduced over age in comparison to young animals (2-way repeated measurements ANOVA, F (2, 2214) = 236.1, *p* < 0.0001, Tukey’s post hoc test: bars indicate significant differences between respective groups in the shown range). (**c**) ABR wave IV amplitude was also significantly reduced over age (2-way repeated measurements ANOVA, F (2, 2025) = 414.7, *p* < 0.0001, Tukey’s post hoc test: bars indicate significant differences between respective groups in the shown range). The middle-aged group had a significantly higher ABR wave IV amplitude between 50 and 75 dB (re thr) than the old animals. For (**b**,**c**): young *n* = 54/27; middle-aged *n* = 28/14; old *n* = 54/27 (ears/animals). Mean ± SEM. (**d**) Antibody against CtBP2/RIBEYE was used as marker for IHC ribbon synapses with afferent auditory neurons. Nuclei were stained with DAPI (blue). Scale bars: 10 μm. (**e**) Immunopositive dots were counted to estimate the number of auditory nerve fiber synapses per IHC, which decreased over age. Arrows indicate a reduced number of CtBP2/RIBEYE-positive dots at the base of IHCs (apical: 1-way ANOVA, F(2, 85) = 11.34; *p* < 0.0001; Bonferroni’s multiple comparisons test: young vs. middle-aged *p* < 0.01; young vs. old *p* < 0.001; middle-aged vs. old *p* > 0.05; medial: 1-way ANOVA, F(2, 88) = 4.61; *p* < 0.05; Bonferroni’s multiple comparisons test: young vs. middle-aged *p* > 0.05; young vs. old *p* < 0.05; middle-aged vs. old *p* > 0.05; midbasal: 1-way ANOVA, F(2, 85) = 11.34; *p* < 0.0001; Bonferroni’s multiple comparisons test: young vs. middle-aged *p* < 0.01; young vs. old *p* < 0.001; middle-aged vs. old *p* > 0.05). Young *n* = 7 mice; middle-aged *n* = 8 mice; old *n* = 7 mice. * *p* < 0.05; ** *p* < 0.01; *** *p* < 0.001. Mean ± SEM.

Aging and acoustic trauma have been shown to induce degeneration of auditory fibers (auditory neuropathy) which damages nerve terminals of the inner hair cell (IHC) (synaptopathy) in mice, non-human primates, and humans [36,37,38]. Auditory-nerve degeneration may occur independently of outer hair cell loss and is called hidden hearing loss [4,10]. To investigate the impact of age on the vulnerability of pre- and post-synaptic structures of the IHC, we analyzed a potential neuropathy by comparing supra-threshold ABR wave amplitudes (Figure 2a) in BLEV mice at different ages. Supra-threshold ABR wave amplitudes change proportionally with discharge rates and the number of synchronously firing auditory fibers [39], the latter of which is defined by the number of IHC synaptic ribbons [40]. Therefore, auditory neuropathy and IHC synaptopathy is well reflected by changes in supra-threshold ABR wave amplitudes, and IHC ribbon numbers, respectively [4,6,34,41].

The auditory stimulus-evoked ABR wave I (Figure 2a,b) reflects the summed activity of the auditory nerve fibers [27] and is a useful functional biomarker of auditory-nerve degeneration after noise exposure [42]. ABR wave IV, on the other hand (Figure 2a,c), reflects the sound-induced activity generated at the level of the IC and lateral lemniscus [27]. The analysis of supra-threshold ABR wave I (Figure 2b) and IV (Figure 2c) revealed a significant reduction of both waves in middle-aged and old BLEV mice compared with young BLEV mice. Although the averaged ABR wave I amplitude of middle-aged BLEV mice was similar to that of old BLEV mice, the ABR wave IV of the former showed a larger amplitude between 50 and 75 dB (re thr) in comparison to old animals, indicating that these animals may compensate a reduced ABR wave I amplitude through a disproportionally enhanced ABR wave IV amplitude.

In young, middle-aged, and old animals we quantified the number of CtBP2/RIBEYE-immuno-positive dots as indicators for ribbon synapses derived from dendrites from spiral ganglion neurons [43] and as an estimation for deafferentation [4] (Figure 2d,e). As especially shown by quantifications of the midbasal cochlear regions (midbasal: >17 kHz [28]) (Figure 2d, right panel) numbers of ribbons in IHCs from young mice were higher than those counted in middle-aged and old mice. This analysis confirmed that the number of CtBP2/RIBEYE-stained ribbon synapses tends to decrease over age (Figure 2e), as also previously observed for aging rats [6].

In conclusion, the auditory input was significantly reduced during the last two thirds of the lifespan of BLEV mice and central output activity varied in middle-aged and old animals.

### 3.3. Central Compensation and Auditory Processing Following Age-Related Reduced Auditory Nerve Activity Differs Depending on Prevailed Latency of Auditory Nerve Response

**Figure 3:**
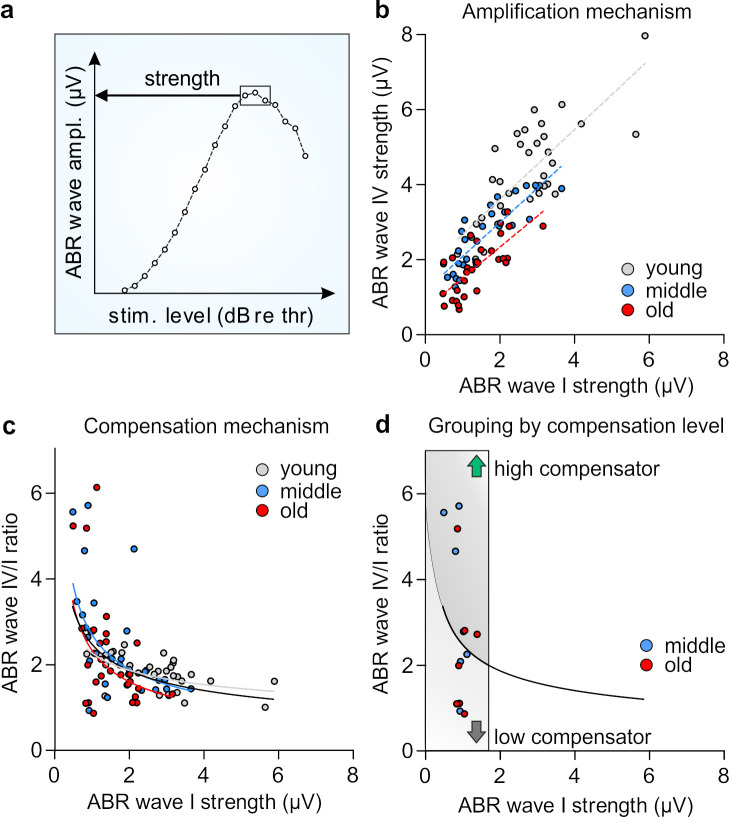
Neural gain mechanisms over age and temporal auditory processing in high and low compensators. (**a**) The ABR wave strength was calculated by averaging the three largest values of wave amplitudes in the I/O function. (**b**) Similar waves I–IV amplification mechanisms (steepness of lines; 2-way ANOVA, F (2, 86) = 0.12, *p* > 0.05, young *n* = 34 animals, middle-aged *n* = 29 animals, old *n* = 29 animals) but decreasing sensitivity over age (*y*-axis intercept; 2-way ANOVA, F (2, 88) = 20.79, *p* < 0.0001). (**c**) Wave IV/I ratio dependent on the wave I amplitude was plotted to picture the central compensation in animals. Regression lines were fitted by power function (y = a*x^b). (**d**) Middle-aged and old animals with reduced wave I (inside grey box; ABR wave I < 1.9 µV) were subdivided along the black regression line (regression of all three groups) into low compensators (dark grey arrow) and high compensators (green arrow). In both groups of age high and low compensators were found.

ABR wave amplitudes and latencies corresponding to click-evoked neuronal activity in the auditory nerve (wave I) and lateral lemiscus and IC (wave IV) were analyzed [27] for increasing stimulus levels. We were interested to what extent the age of animals contributes to the individual’s ability to centrally compensate for reduced auditory nerve activity. We therefore tested the correlation between wave I and wave IV in all age groups. We calculated the *strength* of wave amplitudes in each individual by averaging the three highest amplitude values (Figure 3a). Upon plotting the strength of wave I against the strength of wave IV, we observed a linear correlation between them in all three age groups (Figure 3b). The steepness of regression lines, that represents the degree of wave IV amplification depending on the strength of wave I, was similar in young, middle-aged, and old animals. This indicated that the aging process per se does not affect the physiological amplification mechanism in the ascending auditory pathway. However, the general sensitivity for stimuli, which is determined by *y*-axis intercepts, is decreasing over age (Figure 3b). These results mirror the analysis of ABR waves I and IV amplitude growth functions (Figure 2), which were decreased over age.

To test whether age causes a disproportionally increasing wave IV/I ratio (neural gain), this ratio was calculated for all age groups and the average value between 20 and 80 dB (re thr) was used to plot wave I strength against the wave IV/I ratio (Figure 3c). The power function (y = a*x^b) was inserted as a regression line for each age group and additionally another regression line was plotted for all ears of all groups (black) (Figure 3c). While young animals exhibited a rather flat curve at relatively high values of wave I (Figure 3c, grey), both middle-aged (blue) and old animals (red) showed a wide spreading in the wave IV/I ratio (Figure 3c, *y*-axis) at rather constantly low values of wave I (Figure 3c, *x*-axis). In a subgroup of animals later used for LTP measurements (see below), we also noticed that middle-aged and old animals could be subdivided into two groups (Figure 3d): animals characterized by dots lying below the black regression line were defined by exhibiting a small neuronal gain, which from now on will be called *low compensators*. In contrast, animals reflected by dots above the black trend line exhibited a large neuronal gain, indicating a high central compensation (*high compensators*). Interestingly, individual animals defined as either high (Figure 3d, green arrow) or low compensators (Figure 3d, dark grey arrow), were present in both middle-aged and old mice (Figure 3d, red and blue dots).

**Figure 4:**
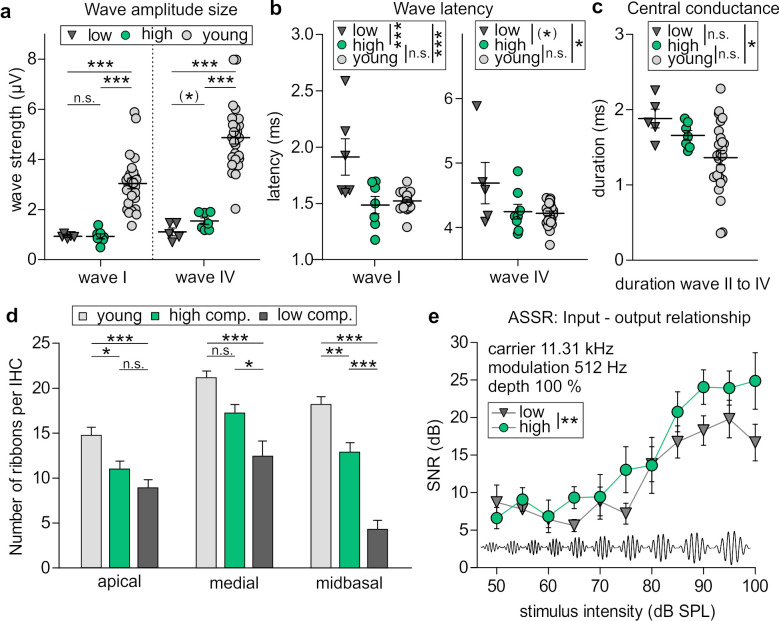
Auditory fine structure analysis and IHC ribbons in high and low compensators. (**a**) ABR wave I strength was similar for high and low compensators and reduced compared with young animals (1-way ANOVA; F(2, 35) = 21.98; *p* < 0.0001; Bonferroni’s multiple comparisons test: high vs. low compensator *p* > 0.05; young vs. high compensator *p* < 0.001; young vs. low compensator *p* < 0.001). ABR wave IV strength was reduced in both ages groups compared to young animals, but high compensators had a trend for a higher ABR wave IV strength compared to low compensators (1-way ANOVA; F(2, 35) = 41.15; *p* < 0.0001; Bonferroni’s multiple comparisons test: high vs. low compensator *p* < 0.1; young vs. high compensator *p* < 0.001; young vs. low compensator *p* < 0.001). (**b**) The latency of both, ABR waves I and IV was similar for high compensators and young animals, while low compensators had a prolonged latency (ABR wave I: 1-way ANOVA; F(2, 36) = 12.55; *p* < 0.0001; Bonferroni’s multiple comparisons test: high vs. low compensator *p* < 0.001; young vs. high compensator *p* < 0.001; young vs. low compensator *p* > 0.05; ABR wave IV: 1-way ANOVA; F(2, 35) = 4.592; *p* < 0.05; Bonferroni’s multiple comparisons test: high vs. low compensator *p* < 0.1; young vs. high compensator *p* < 0.05; young vs. low compensator *p* > 0.05). (**c**) Low, but not high compensators had a prolonged conduction of central processing, measured by duration between ABR waves II and IV (1-way ANOVA, F(2, 34) = 4.045; *p* < 0.05; Bonferroni’s multiple comparisons test: high vs. low compensator *p* > 0.05; young vs. high compensator *p* > 0.05; young vs. low compensator *p* < 0.05). For (**a**–**c**): Low compensators *n* = 5; high compensators *n* = 7; young *n* = 26 animals. (**d**) While high compensators showed only moderate reduction of IHC ribbons in the apical and midbasal turns when compared to young animals, low compensators showed a highly significant reduction of ribbons in all three cochlear turns in comparison with young animals, which was most prominent for high-frequency cochlear regions (apical: 1-way ANOVA, F(2, 34) = 9.1; *p* < 0.001; Bonferroni’s multiple comparisons test: high vs. low compensator *p* > 0.05; young vs. high compensator *p* < 0.05; young vs. low compensator *p* < 0.001; medial: 1-way ANOVA, F(2, 35) = 9.72; *p* < 0.001; Bonferroni’s multiple comparisons test: high vs. low compensator *p* < 0.05; young vs. high compensator *p* > 0.05; young vs. low compensator *p* < 0.001; midbasal: 1-way ANOVA, F(2, 35) = 41.93; *p* < 0.0001; Bonferroni’s multiple comparisons test: high vs. low compensator *p* < 0.001; young vs. high compensator *p* < 0.01; young vs. low compensator *p* < 0.001). Young *n* = 4 mice; low compensators *n* = 5 mice; high compensators *n* = 5 mice. (**e**) Input–output relationship of auditory steady state responses with a carrier frequency of 11.32 kHz, a modulation frequency of 512 Hz, and a modulation depth of 100% showed a reduction of temporal auditory resolution in low compensators (2-way ANOVA, F(1, 106) = 7.52, *p* < 0.01; Bonferroni’s multiple comparisons test for all stimulus intensities: *p* > 0.05). Low compensators *n* = 5; high compensators *n* = 7. (*) *p* < 0.1; * *p* < 0.05; ** *p* < 0.01; *** *p* < 0.001. Mean ± SEM.

High and low compensators did not differ in the strength of ABR wave I which was reduced in both of these groups compared to young animals (Figure 4a, left panel).

In contrast, the strength of ABR wave IV in high compensators had a trend for a higher level compared to low compensators, while both were significantly reduced in comparison to young animals (Figure 4a, right panel), indicating that high and low compensators differed in central compensation rather than in the size of the auditory nerve amplitude. Strikingly, however, the latency of ABR waves I and IV was significantly prolonged in low compensators in comparison to high compensators and young animals (Figure 4b). Taking into account that, at any given characteristic frequency in the auditory system, high-SR fibers with low thresholds have the shortest latencies in comparison to fibers with low-spontaneous firing rates and high thresholds [44,45], our findings suggest that low compensators exhibit compromised high-SR auditory fiber processing.

Additional to prolonged latency of ABR wave I in low compensators, the duration from ABR wave II to ABR wave IV was longer in low compensators compared to young BLEV mice (Figure 4c), indicating that not only the auditory nerve response is delayed but also the central conductance is prolonged in these animals.

Next, we were interested if high and low compensators also differ in their number of IHC ribbons. Both high and low compensators had reduced numbers of IHC ribbons (Figure 4d). However, the decline was much higher in low compensators (Figure 4d). Especially in high-frequency cochlear regions the difference between high and low compensators was most prominent and ribbon loss in low compensators exceeded > 50% reduction, indicating a significant contribution of high-SR auditory fibers (Figure 4d, midbasal).

We finally searched for a difference in temporal processing between high and low compensators using ASSR as described [6], using the amplitude of a carrier frequency of 11.32 kHz that was modulated by a second, slower frequency of 512 Hz with a modulation depth of 100%. Especially for high SPLs > 60 dB a significant attenuated temporal resolution of auditory stimuli was observed in low compensators in comparison to high compensators (Figure 4e). This suggests that the reduced strength of ABR wave IV (Figure 4a, right panel) and the prolonged latency of ABR wave I and ABR wave IV in low compensators (Figure 4b), as well as reduced number of IHC ribbons (Figure 3d) may be linked to a lower temporal auditory resolution in this group.

Conclusion: We observed that both middle-aged and old animals could be subdivided in groups with a lower and higher ability to centrally compensate reduced cochlear synaptopathy. Auditory nerve activity (ABR wave I), although not different in ABR wave size, was prolonged in latency and central conduction time in low compensators in comparison to high compensators and young animals. Furthermore, compared with high compensators, the number of IHC ribbons was strongly reduced in high-frequency regions of low compensators. All of these findings were linked to an attenuated ASSR to follow amplitude-modulated tones in low compensators. This suggests that over age the ability of the brain to disproportionally elevate output activity relative to cochlear input activity (neural gain) and temporal auditory processing is compromised when fast auditory processing is diminished.

### 3.4. Delayed Auditory Nerve Response and Attenuated Central Auditory Processing Due to Age-Dependent Reduced Auditory Nerve Activity Is Linked with Lower Hippocampal Long-Term Potentiation

We previously observed that central compensation of cochlear synaptopathy is compromised when a critical diminution of auditory input, encompassing high-SR auditory fibers, hampers hippocampal field excitatory postsynaptic potentials (fEPSPs) [24]. Hypothesizing differences in memory-linked facilitation pathways between low and high compensating groups, LTP was measured as described [24]. The recording electrode was placed in the stratum radiatum (SR) of the CA1 region, while stimulating the CA3 Schaffer’s collateral axons which have synaptic contacts to CA1 pyramidal cells [24]. LTP was induced in acute coronal brain slices of young, middle-aged, and old BLEV mice. LTP recordings from middle-aged and old animals were then subdivided according to high and low compensators, regardless of their age.

**Figure 5:**
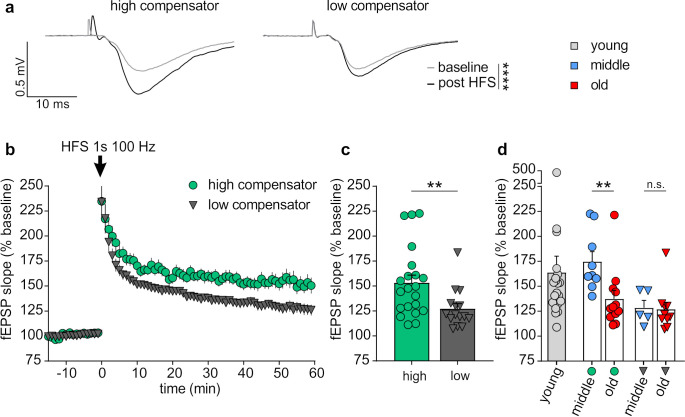
Long-term potentiation (LTP)-induced changes of field excitatory postsynaptic potentials (fEPSPs) in low and high compensators. (**a**) Representative traces before (baseline) and after (post high frequency stimulation) LTP induction as well as (**b**) averaged time courses of fEPSP slopes in acute coronal brain slices displayed prominent LTP in all groups of animals (baseline vs. post HFS; high compensators: Mann–Whitney U test, U (21) = 0, *p* < 0.0001; low compensators: Mann–Whitney U test, U (15) = 0, *p* < 0.0001). (**c**) Highly compensating BLEV mice (153.38% ± 7.56%; *n* = 7/21 animals/slices) showed significantly elevated LTP in comparison to low compensating (127.39% ± 5.05%; *n* = 5/15 animals/slices) BLEV mice (Mann–Whitney U test, U (21, 15) = 74, *p* < 0.01). (**d**) Highly compensating animals are present in both, middle-aged (174.69% ± 9.96%; *n* = 4/9 animals/slices) and old (137.39% ± 11.08%; *n* = 3/9 animals/slices) groups and do not differ significantly from young animals (163.84% ± 16.31%; *n* = 7/21 animals/slices; Kruskal–Wallis test with two-stage linear step-up procedure of Benjamini, Krieger, and Yekutieli: young vs. middle-aged high compensator, *p* > 0.05; young vs. old high compensator, *p* > 0.05). At the same time, we observed significant differences between highly compensating middle-aged (174.69% ± 9.96%; *n* = 4/9 animals/slices) and highly compensating old animals (137.39% ± 11.08%; *n* = 3/9 animals/slices), but no prominent difference between low compensating middle-aged (128.35% ± 7.46%; *n* = 2/5 animals/slices) and low compensating old animals (126.90% ± 6.86%; *n* = 3/10 animals/slices; Kruskal–Wallis test with two-stage linear step-up procedure of Benjamini, Krieger, and Yekutieli: highly compensating middle-aged vs. highly compensating old, *p* < 0.01; low compensating middle-aged vs. low compensating old, *p* > 0.05). ** *p* < 0.01; *** *p* < 0.001. Mean ± SEM. In (**c**,**d**) each dot represents fEPSP slope (average of last 10 min post HFS) of a single coronal brain slice.

LTP was induced by high-frequency stimulation (HFS; 1s, 100 Hz) and the mean of the last 10 min from the 60 min recording showed that the post HFS fEPSPs were significantly different from the baseline in both low and highly compensating animals (Figure 5a,b).

However, animals that showed poor central compensation of cochlear synaptopathy (Figure 4d), had significantly reduced hippocampal LTP maintenance compared to animals with a higher capacity for central compensation (Figure 5c). Subdivision of high and low compensators according to age (Figure 5d) revealed that for high compensators age still plays a role, as middle-aged high compensators (blue circles) had a significantly higher LTP than old high compensators (Figure 5d; red circles). In contrast, for low compensators no difference between middle-aged (Figure 5d; blue triangles) and old animals (Figure 5d; red triangles) could be observed (Figure 5d). Interestingly, the LTP in middle-aged high compensators was not different from that of young control animals (Figure 5d; light grey).

Importantly, neither high nor low compensators exhibited changes in basal synaptic transmission compared to young animals (Figure S1a) as both highly and low compensating groups displayed a similar growth of fEPSP slopes (Figure S1b), but shifted slightly in fiber volley amplitude changes (Figure S1c). However, a regression between both (fEPSP slopes and fiber volley amplitudes) was not different (Figure S1d). This indicates that no changes in presynaptic function occurred which could have caused the observed differences, and thus point to normal activity in pre-synaptic Schaffer’s collaterals in both highly and low compensating groups. Moreover, paired-pulse facilitation (PPF) was similar between both groups of animals (Figure S2a–c). Additionally, both high and low compensators’ PPF was similar to that of young animals at almost every interpulse interval applied (Figure S2b,c) with the exception of the paired-pulse ratio of the EPSP2/EPSP1 amplitude observed at 10 ms (Figure S2c). This implies that rather than diminished presynaptic fEPSPs and deficient short-term plasticity, attenuated postsynaptic fEPSPs and LTP is linked to reduced central compensation in low compensating groups in comparison to high compensators.

Conclusively, this finding suggests that low compensators with delayed and reduced auditory nerve response and attenuated ability to respond with central compensation and temporal precision exhibit a less pronounced ability to recruit hippocampal LTP in comparison to high compensators with an auditory nerve response which is not delayed.

### 3.5. Delayed Auditory Nerve Response and Attenuated Central Auditory Processing Due to Age-Dependent Reduced Auditory Nerve Activity Is Linked to Lower Levels of Hippocampal BDNF

**Figure 6:**
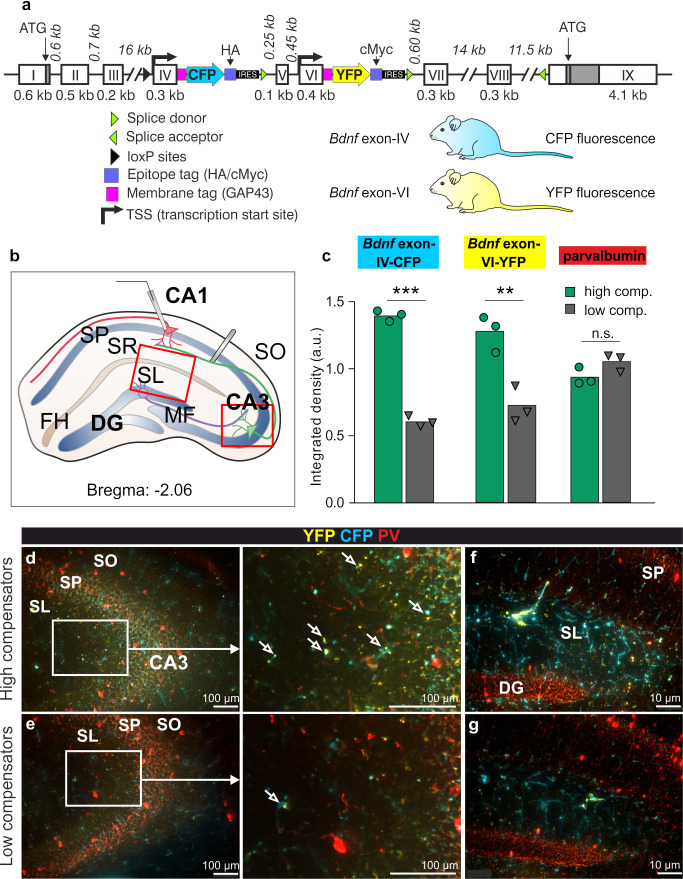
*Bdnf* exon IV/VI and PV expression in the hippocampus of low and high compensators. (**a**) *Bdnf* gene construct depicting insertion sites of the BLEV construct. (**b**) Abstract scheme of hippocampal coronal section. Red boxes indicate the inset seen in d/e (CA3 region) or f/g (SL/DG). SL = stratum lucidum; DG = dentate gyrus; SP = stratum pyramidale; SO = stratum oriens; FH = fissura hippocampalis; SR = stratum radiatum; MF = mossy fiber. (**c**) Quantification of SL/DG region shows larger CFP (Mann–Whitney U test; U(4) = 24.31; *p* < 0.0001) and YFP (Mann–Whitney U test; U(4) = 4.994; *p* = 0.0075) expression in high compensators but no difference of PV in both groups (Mann–Whitney U test; U(4) = 2.127; *p* > 0.05). High compensator *n* = 3/6; low compensator *n* = 3/6 (animals/hippocampal hemispheres). ** *p* < 0.01; *** *p* < 0.001. Bars represent Mean. (**d**) Highly compensating animal showing strong expression of *Bdnf* exon IV (CFP) and *Bdnf* exon VI (YFP) in the hippocampal CA3 region (upper panel). With higher magnification, a colocalization of CFP and YFP could be observed (middle panel). (**e**) A low compensating animal showed reduced expression of CFP and YFP in the hippocampal CA3 region (upper and middle panel) compared to high compensators. (**f**) In the SL, a prominent CFP and YFP expression could also be observed in the high compensators, (**g**) which was reduced in the low compensator animals.

Attenuated central compensation following crucial diminution of auditory input was previously shown not only to hamper LTP, but was also linked to diminished recruitment of *Bdnf* exon-IV-CFP and exon-VI-YFP transcripts in the hippocampus [24]. To test if low and high compensators differ in recruitment of activity-dependent *Bdnf* transcripts, we analyzed activity-dependent changes of CFP and YFP, tagged via bi-cistronic expression to translational sites of *Bdnf* exon-IV and -VI mRNAs in BLEV reporter mice, as described previously (see introduction, Figure 6a, [26]).

We examined *Bdnf* exon-IV-CFP and exon-VI-YFP in deconvoluted high resolution fluorescence stacks in the hippocampus (red framed schematic view in Figure 6b). Stacks were costained with parvalbumin (PV) used to identify changes in fast-spiking GABAergic interneurons [24,46]. Among one pair of middle-aged high and low compensators and two pairs of old high and low compensators tested, all high compensators expressed significantly higher levels of *Bdnf* exon-IV-CFP and exon-VI-YFP (Figure 6c, averaged for both age groups, each performed in duplicates). At low magnification of the hippocampal CA3 region, higher *Bdnf* exon-IV-CFP and exon-VI-YFP expression could be observed in high compensators (Figure 6d) compared to low compensators (Figure 6e). Higher magnification revealed the presence of more abundantly overlapping CFP and YFP puncta in the stratum lucidum (SL) of high compensators in comparison to low compensators (Figure 6d, lower panel). For the SL, between the CA1 region and dentate gyrus (DG) (Figure 6b, frame around SL), numerous YFP-positive *Bdnf* exon-VI transcripts were translated in nerve terminals [26] close to CFP-positive *Bdnf* exon-IV transcripts expressed in capillaries [26], particularly in high compensators (Figure 6f) in comparison to low compensators (Figure 6g). So far, no differences in the levels of PV labeling were seen between low and high compensators at any age. Further quantifications of higher magnification images may be required to elucidate if more subtle differences in labeling of PV-positive staining may be identified at perisomatic or axo-dendritic positions between low and highly compensating groups.

Conclusively, in both, middle-aged and old animals, *Bdnf* transcript-IV/VI levels were diminished in low compensating groups in comparison to high compensators (Figure 6).

**Figure 7:**
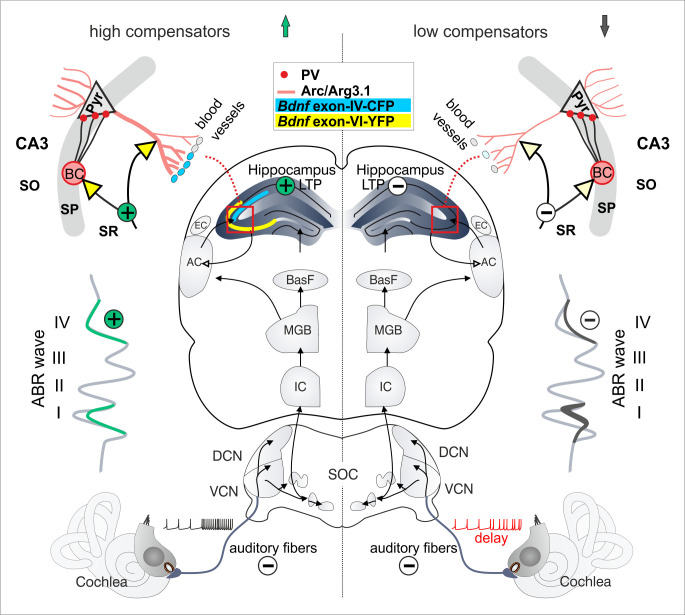
Abstract scheme of mechanisms in the auditory pathway caused by central compensation. High compensators (left side) show disproportionally increased ABR wave IV despite reduced ABR wave I. This is reflected by intact LTP and prominent expression of CFP (*Bdnf* exon-IV) and YFP (*Bdnf* exon-VI). Low compensators (right side) cannot overcome the reduced ABR wave I and therefore have also a reduction in ABR wave IV. Furthermore, LTP is decreased, and CFP and YFP expression is diminished. The black arrows represent the ascending auditory pathway, beginning in the cochlea, from where the signal is sent via auditory fibers to the auditory brainstem (DCN, VCN), midbrain (IC) and the medial geniculate body (MGB), from where it is directly sent to the cortex (AC) and indirectly via the basal forebrain (BasF) to the hippocampus.

Overall the findings suggest that independently from an animal being middle-aged or old, some animals with a delayed and reduced auditory nerve response exhibited a lower central neural gain (Figure 7, right panel, ABR wave, low compensators) in comparison to those with a response that was not delayed and a higher central neural gain (Figure 7, left panel, ABR wave, high compensators). These low compensators exhibited an attenuated sensitivity to follow amplitude-modulated tones (Figure 7, ABR wave, minus), generated less pronounced LTP response (Figure 7, hippocampus, minus) and diminished levels of *Bdnf* exon-VI-YFP and *Bdnf* exon-IV-CFP transcripts close to capillaries in hippocampal regions (Figure 7, yellow, cyan hippocampus and inset).

## 4. Discussion

We here describe that independently from being middle-aged or old and unrelated to an overall reduced maximal auditory nerve amplitude size, delayed auditory nerve activity is linked to a lower central compensation and temporal coding capacity and a reduced ability to recruit LTP and hippocampal *Bdnf* transcripts. This indicates that not age-dependent cochlear synaptopathy per se, but attenuation of fast (high-SR) auditory processing, may limit memory-dependent temporal auditory precision and thereby contribute to age-dependent deficits in speech understanding in the presence of noise.

### 4.1. Auditory (Temporal) Processing Deficits Due to Age-Dependent Cochlear Synaptopathy Differ Depending on Prevailed Latency of Auditory Nerve Response

Age-related hearing loss has long been discussed in the context of problems of understanding speech in the presence of background noise (see [47,48,49] for a review). Even when elderly listeners retained near-normal audiometric thresholds, hearing difficulties in acoustically complex environments have been reported (e.g., [17,18,19,49]). Moreover, recent studies suggest that already before hearing loss is measurable in a standard clinical audiogram, a reduction of supra-threshold stimuli encoding precision can be observed that may be linked to a cochlear neuropathy [8,9,50] and may cause poor speech discrimination [2]. Until now fibers with a low SR and a high threshold were suggested to be responsible for temporal processing deficits [5,10], as (i) they emerge perceptually at supra-threshold levels and in challenging listening situations such as in the presence of background noise [51], providing a rationale for the numerous studies that report listening deficits in humans despite normal hearing thresholds (e.g., [5,10,17,18,19]). High-SR auditory fibers would be driven to saturation under these conditions [2,12,13], as envelope cues that are important for speech-on-speech masking release rely particularly on low-SR supra-threshold coding [52], and (ii) low-SR fibers were also shown to have a high vulnerability to noise and aging [10,14,15,16]. However, recently some conflicting findings which debate the hypothesis that low-SR linked cochlear synaptopathy is the main source of temporal auditory resolution were reported. Thus, despite similar peripheral sensitivity between young and middle-aged groups a loss of temporal resolving power with reduced speech understanding was found starting from middle-age [48]. In line with this observation various studies could not find a direct association between a potential substrate of cochlear synaptopathy and deficits in the detection of proper envelope time cues in humans [23,53,54,55].

The delay in auditory nerve response in the group of low compensating animals observed in the present study that is linked with a significantly increased ribbon loss in high-frequency cochlear turns compared to high compensators (Figure 4), suggests that a critical proportion of high-SR auditory fibers, shown to account > 60% of auditory fibers [56], may be affected. High-SR auditory fibers are known to be responsible for the shortest latencies of auditory responses at any given characteristic frequency [44]. This, therefore, may best explain the delayed auditory nerve response and prolonged central conductance (Figure 3) observed in low compensators in comparison to high compensators. High-SR auditory fibers mature after hearing onset with fast auditory processing by developing active feedforward and feedback PV+ interneuron microcircuits providing thereby the basis of auditory-specific connectivity to fronto-striatal brain regions responsible for contrast-amplification and neural gain (see [57] for a review). This suggests that a crucial attenuation of high-SR auditory fiber activity, as here suggested for low-compensating groups, might be functionally linked to the reduced central compensation in these groups (Figure 4). Indirectly the requirement for sustained numbers of high-SR fibers with low thresholds for central compensation of synaptopathy was already predictable from a computerized model that argued that the persistence of a crucial level of high-SR fibers with low thresholds after acoustic noise trauma is a prerequisite for the generation of a sufficient homeostatic increase in discharge rate in auditory brainstem neurons that are targeted by auditory fibers [58]. Within this view a crucial level of maintained spike trains in auditory fibers is essential to drive compensating increases in spike trains in target neurons after deprived auditory input [58,59]. Regarding this scenario, the higher firing rates in auditory brainstem regions following deafferentation may be driven by a homeostatic decrease in inhibition, as shown to occur in numerous studies following hearing loss [60,61,62,63]. This enhanced neural activity is likely to be amplified through cholinergic memory-dependent facilitation circuit (see below). Only previously these central compensation circuits that are generated through neural gain were suggested as a prerequisite for sustained temporal auditory processing following cochlear synaptopathy over age [6].

Conclusively, independently from age and size of the auditory nerve amplitude, lower and higher central compensation (with a correspondingly lower or higher temporal resolution) is linked to delayed or normal auditory nerve response and more or less IHC ribbon loss, respectively. Different kinds of cochlear synaptopathy linked to differential power of the brain to centrally compensate for auditory deprivation, must, therefore, be interpreted in the context of proper central auditory processing over age.

### 4.2. Auditory (Temporal) Processing Deficits Due to Age-Dependent Cochlear Synaptopathy Differ Depending on Hippocampal LTP and Bdnf Transcript Recruitment

We here report that independently of being middle-aged or old, animals with prolonged auditory nerve latencies exhibit reduced central auditory compensation, lower hippocampal LTP levels and lower recruitment of *Bdnf* transcripts in hippocampal regions in comparison to animals with prevailed auditory nerve activity. Central auditory compensation is a complex process that, similar to the attention-driven contrast amplification of auditory responses, has only previously been suggested to involve coactivation of auditory and fronto-striatal regions such as (i) the basal forebrain to accentuate particular auditory stimuli [64,65,66], (ii) the inferior frontal gyrus activity, to distinguish new or deviant signals from previous ones [67,68], (iii) the hippocampus to extract and memorize the behaviorally relevant signal and to adjust synaptic strength [64,65,69], and (iv) prefrontal cortex regions to balance attention-driven plasticity responses [70,71,72].

During this process, auditory information, processed in the medial geniculate body, can activate the network of fronto-striatal brain areas [65,66,71]. Although not explicitly shown for age-dependent hearing loss, previous and present studies demonstrating central neural gain following age-dependent cochlear synaptopathy ([6], present study), may suggest that increased spontaneous and evoked activity in the cochlear nucleus, as shown to occur after acoustic trauma [24,63,73,74,75,76,77,78], can be amplified through an attention- and learning-dependent circuit. Central neural gain thus likely requires critical high-SR auditory fiber power that maintains a proper baseline of PV+ interneuron-dependent microcircuits (review [57]), on the basis of which central compensating adjustment and contrast-amplification processes occur, the latter of which are crucial for listeners to properly attend to relevant stimuli, while ignoring irrelevant ones [57,79,80,81,82,83].

As shown for contrast amplification pathways [71], these central amplification processes following auditory deprivation are suggested to require a context-specific signal, to assure that synchronized output responses (neural gain) occur specifically in the frequency-deprived regions [24]. Thus, activity-dependent activation of *Bdnf* transcripts was previously suggested as a context-specific carrier [24]. Accordingly, following mild acoustic trauma or sound enrichment, increased levels of *Bdnf* exon-IV-CFP and -VI-YFP were observed in the auditory brainstem and hippocampus that correlated with enhanced or compensated central neural gain and enhanced hippocampal LTP levels [24,84]. In contrast, upon a trauma-induced reduction of auditory input that comprised critical high-SR auditory fiber contribution, hippocampal LTP and *Bdnf* transcript recruitment was diminished similar to central neural gain [24].

In line with this, in the present study a crucial reduction of auditory input in low compensating aged groups was accompanied by lower hippocampal LTP levels and lower recruitment of *Bdnf* transcripts in hippocampal regions compared to same aged high compensators.

Interestingly, this effect was not only independent of age (Figure 3c,d) but also independent of short term plasticity changes, measured by PPF (Figure S2). This may suggest that the long-term consolidation process of memory that e.g., requires activity-dependent BDNF and glucocorticoid receptor-triggered spine formation to form and maintain learning-dependent synapses [85], rather is a part of a central neural gain processes and subsequent to prevailed fast (high-SR) temporal coding.

Considering how *Bdnf* transcript recruitment may act during facilitation circuits, it is interesting to take into account that the increased levels of *Bdnf* exon-VI-YFP fluorescence in high-compensator groups (Figure 6) were most likely observed in presynaptic nerve endings. This is concluded from studies that showed that BDNF was targeted via anterograde transport in an activity-dependent way to mossy fiber nerve terminals [86] and from colocalization of *Bdnf* exon-VI-YFP with vesicular glutamate transporters in nerve terminals of pyramidal neurons [26]. Low compensating groups showed less *Bdnf* exon-IV-CFP expression in capillary vessels within the highly vascularized fissura hippocampalis region (Figure 6). *Bdnf* exon-IV was traced in these regions to platelets [26], where it was suggested to respond to the activity of store-operated calcium channels [87]. Regarding that specific neuronal activity may tightly regulate blood flow [88], we may hypothesize that the link between reduced recruitment of activity-driven *Bdnf* exon-VI transcripts in nerve endings and of *Bdnf* exon-IV-CFP expression in capillary vessels in low compensators in comparison to high compensators (Figure 6), may reflect a lower ability of neuro-vascular coupling, a feature that requires more extensive future studies. It is also important to point out that temporal auditory processing was not only shown to rely on speed and working memory [25,55,89,90], but also that hypertension-linked memory-deficits over age were linked with lower BDNF levels [91] and that attention- and memory-linked accentuation processes can fail due to deficits in age-dependent circulation of blood flow [92].

Within this context, hearing loss, which is linked to an increased risk of cognitive decline in epidemiological studies [93,94,95], may reflect the fragility of an individual’s neural substrates, particularly of those that control fast (high-SR) auditory processing and auditory-modality specific recruitment of LTP-BDNF-dependent processing, rather than hearing loss, cochlear synaptopathy or age per se.

## Figures and Tables

**Table 1:** Statistical information of the results.

Figure	Comparison	Statistical Test	Test Value	*p*-Value	Post-Hoc Test with *p*-Value		*n*—Number
					**Tukey´s multiple comp. Test**		
Fig. 1a	Click-ABR	1-way ANOVA	F (2, 157) = 39.70	*p* < 0.0001	Y vs. M-A	*p* > 0.05	Y *n* = 27 animals M-A *n* = 14 animals O *n* = 27 animals
					Y vs. O	*p* < 0.0001	
					MA vs. O	*p* < 0.0001	
Fig. 1b	Noise-ABR	1-way ANOVA	F (2, 157) = 27.23	*p* < 0.0001	Y vs. M-A	*p* > 0.05	
					Y vs. O	*p* < 0.0001	
					M-A vs. O	*p* < 0.0001	
Fig. 1c	f-ABR	2-way ANOVA	F (2, 741) = 73.33	*p* < 0.0001	Y vs. M-A	*p* < 0.05 for all freq > 22.6 kHz	
					Y vs. O	*p* < 0.05 for all freq shown	
					M-A vs. O	*p* < 0.05 for all freq shown except 22.6 kHz	
					**Tukey´s multiple comp. Test**		
Fig. 2b	ABR wave I ampl.	2-way ANOVA	F (2, 2214) = 236.1	*p* < 0.0001	Y vs. M-A	*p* < 0.05 for all SPL > 20 dB re Thr	Y *n* = 24 animals M-A *n* = 25 animals O *n* = 22 animals
					Y vs. O	*p* < 0.05 for SPL between 15 and 85 dB re Thr	
					M-A vs. O	*p* > 0.05	
Fig. 2c	ABR wave IV ampl.	2-way ANOVA	F (2, 2025) = 414.7	*p* < 0.0001	Y vs. M-A	*p* < 0.05 for all SPL > 15 dB re Thr	
					Y vs. O	*p* < 0.05 for all SPL > 20 dB re Thr	
					M-A vs. O	*p* < 0.05 for SPL between 50 and 75 dB re Thr	
Fig. 2e	IHC ribbons apical	1-way ANOVA	F (2, 85) = 11.34	*p* < 0.0001	Y vs. M-A	*p* < 0.01	Y *n* = 7 animals M-A *n* = 8 animals O *n* = 7 animals
					Y vs.O	*p* < 0.001	
					M-A vs. O	*p* > 0.05	
	IHC ribbons medial	1-way ANOVA	F (2, 88) = 4.61	*p* = 0.0125	Y vs. M-A	*p* > 0.05	
					Y vs. O	*p* < 0.05	
					M-A vs. O	*p* > 0.05	
	IHC ribbons midbasal	1-way ANOVA	F (2, 85) = 11.34	*p* < 0.0001	Y vs. M-A	*p* < 0.01	
					Y vs. O	*p* < 0.001	
					M-A vs. O	*p* > 0.05	
Fig. 3b	Amplification Y	Regression	y = 0.9322x + 1.7392	R^2^ = 0.6005			Y *n* = 34 animals M-A *n* = 29 animals O *n* = 29 animals
	Amplification M-A		y = 0.9081x + 1.1758	R^2^ = 0.7313			
	Amplification O		y = 0.8248x + 0.6833	R^2^ = 0.5378			
	Comparison between ages	Steepness of regression lines	F (2, 86) = 0.12	*p* = 0.883			
		Are regression lines different?	F (2, 88) = 20.79	*p* < 0.0001			
Fig. 3c	Compensatio*n* Y	Regression	y = 2.2714x^−0.277^	R^2^ = 0.2768			
	Compensatio*n* M-A		y = 2.3313x^−0.532^	R^2^ = 0.3446			
	Compensatio*n* O		y = 2.6943x^−0.502^	R^2^ = 0.2068			
	Compensatio*n* all		y = 2.4733x^−0.405^	R^2^ = 0.2815			
					**Bonferroni’s multiple comp. test**		
Fig. 4a	ABR wave I strength	1-way ANOVA	F (2, 35) = 21.98	*p* < 0.0001	HC vs. LC	*p* > 0.05	LC *n* = 5 HC *n* = 7 Y *n* = 26 animals mean of both ears
					Y vs. HC	*p* < 0.001	
					Y vs. LC	*p* < 0.001	
	ABR wave IV strength	1-way ANOVA	F (2, 35) = 41.15	*p* < 0.0001	HC vs. LC	*p* < 0.1	
					Y vs. HC	*p* < 0.001	
					Y vs. LC	*p* < 0.001	
Fig. 4b	ABR wave I latency	1-way ANOVA	F (2, 36) = 12.55	*p* < 0.0001	HC vs. LC	*p* < 0.001	
					Y vs. HC	*p* < 0.001	
					Y vs. LC	*p* > 0.05	
	ABR wave IV latency	1-way ANOVA	F (2, 35) = 4.592	*p* = 0.0169	HC vs. LC	*p* < 0.1	
					Y vs. HC	*p* < 0.05	
					Y vs. LC	*p* > 0.05	
Fig. 4c	Central conductance	1-way ANOVA	F (1, 34) = 4.045	*p* = 0.0266	HC vs. LC	*p* > 0.05	
					Y vs. HC	*p* > 0.05	
					Y vs. LC	*p* < 0.05	HC *n* = 7 LC *n* = 6
Fig. 4d	IHC ribbons apical	1-way ANOVA	F (2, 34) = 9.1	*p* < 0.001	HC vs. LC	*p* > 0.05	Y *n* = 7 animals M-A *n* = 8 animals O *n* = 7 animals
					Y vs. HC	*p* < 0.05	
					Y vs. LC	*p* < 0.001	
	IHC ribbons medial	1-way ANOVA	F (2, 35) = 9.72	*p* < 0.001	HC vs. LC	*p* < 0.05	
					Y vs. HC	*p* > 0.05	
					Y vs. LC	*p* < 0.001	
	IHC ribbons midbasal	1-way ANOVA	F (2, 35) = 41.93	*p* < 0.0001	HC vs. LC	*p* < 0.001	
					Y vs. HC	*p* < 0.01	
					Y vs. LC	*p* < 0.001	
Fig. 4e	ASSR input–output function	2-way ANOVA	F (1, 106) = 7.52	*p* = 0.0072	HC vs. LC	*p* > 0.05	HC *n* = 7 LC *n* = 6
Fig. 5b	HC baseline vs. post HFS	Mann–Whitney U	U (21) = 0	*p* < 0.0001			*n* = animals/slices Y *n* = 7/21 HC *n* = 7/21 LC *n* = 5/15
	LC baseline vs. post HFS		U (15) = 0	*p* < 0.0001			
Fig. 5c	LTP HC vs. LC	Mann–Whitney U	U (21, 15) = 74	*p* = 0.0066			
					**Two-stage linear step-up procedure of Benjamini, Krieger, and Yekutieli**		
Fig. 5d	LTP high and LC dependent on age and Y control group	1-way nonparametric ANOVA on ranks (Kruskal–Wallis test)	H (5) = 20.18, *p* = 0.0005	*p* < 0.001	HC M-A vs. O	*p* < 0.01	
					low comp. M-A vs. O	*p* > 0.05	
					Y vs. M-A HC	*p* > 0.05	
					Y vs. M-A LC	*p* > 0.05	
					Y vs. O HC	*p* > 0.05	
					Y vs. O LC	*p* < 0.01	
					M-A HC vs. LC	*p* < 0.01	
					O HC vs. LC	*p* > 0.05	
Fig. 6c	*Bdnf* exon-IV-CFP	Mann–Whitney U	U (4) = 24.31	*p* < 0.0001			
	*Bdnf* exon-IV-YFP		U (4) = 4.994	*p* = 0.0075			
	Parvalbumin		U (4) = 2.127	*p* = 0.1005			
Fig. S1b	IOR fEPSP slope	2-way ANOVA	F (2, 300) = 1.446	*p* = 0.2371			
					**Two-stage linear step-up procedure of Benjamini, Krieger, and Yekutieli**		*n* = animals/slices Y *n* = 7/21 HC *n* = 7/20 LC *n* = 5/12
Fig. S1c	IOR fiber volley amplitude	2-way ANOVA	F (2, 300) = 4.127	*p* = 0.0171	HC vs. LC	*p* > 0.05	
					Y vs. HC	*p* > 0.05	
					Y vs. LC	*p* > 0.05	
Fig. S1d	fEPSP slope vs. fiber volley amplitude	Difference between regression lines (slopes)	F (2, 323) = 0.69	*p* = 0.5023			
Fig. S2b	Paired-pulse ratio EPSP2/EPSP1 (slope)	2-way ANOVA	F (2, 330) = 0.9445	*p* = 0.3899			*n* = animals/slices Y *n* = 7/21 HC *n* = 7/20 LC *n* = 5/16
					**Two-stage linear step-up procedure of Benjamini, Krieger, and Yekutieli**		
Fig. S2c	Paired-pulse ratio EPSP2/EPSP1 (amplitude)	2-way ANOVA	F (2, 330) = 4.487	*p* = 0.0120	HC vs. LC	*p* > 0.05	
					Y vs. HC	*p* > 0.05	
					Y vs. LC (only at 10 ms interpulse interval)	*p* < 0.01	

Y = young, M-A = middle-aged, O = old, HC = high compensator, LC = low compensator.

## Supplementary Materials

The following are available online at https://www.mdpi.com/2076-3425/10/10/710/s1, Figure S1: Input-output relationship between fEPSP slope, fiber volley amplitude and stimulus intensity, Figure S2: Paired-pulse facilitation (PPF) as an indicator of short-term plasticity.

## Abbreviations

Auditory brainstem responses (ABR); artificial cerebrospinal fluid (ACSF); analysis of variance (ANOVA); auditory steady state responses (ASSR); BDNF-live-exon-visualization (BLEV); cyan- and yellow-fluorescent-protein (CFP/YFP); C terminal-binding protein 2 (CtBP2); dentate gyrus (DG); field excitatory postsynaptic potentials (fEPSPs); high-frequency stimulation (HFS); high spontaneous firing rate (high-SR); inferior colliculus (IC); inner hair cell (IHC); input–output relationship (IOR); low spontaneous firing rate (low-SR); long-term potentiation (LTP); paired-pulse facilitation (PPF); parvalbumin (PV); relative to threshold (re thr); standard error of the mean (SEM); standard deviation (SD); stratum lucidum (SL); stratum radiatum (SR).

## References

[1] Fullgrabe C., Moore B.C. (2014). Effects of age and hearing loss on stream segregation based on interaural time differences. J. Acoust. Soc. Am..

[2] Bharadwaj H.M., Verhulst S., Shaheen L., Liberman M.C., Shinn-Cunningham B.G. (2014). Cochlear neuropathy and the coding of supra-threshold sound. Front. Syst. Neurosci..

[3] Bramhall N., Ong B., Ko J., Parker M. (2015). Speech perception ability in noise is correlated with auditory brainstem response wave I amplitude. J. Am. Acad. Audiol..

[4] Kujawa S.G., Liberman M.C. (2009). Adding insult to injury: Cochlear nerve degeneration after “temporary” noise-induced hearing loss. J. Neurosci..

[5] Sergeyenko Y., Lall K., Liberman M.C., Kujawa S.G. (2013). Age-related cochlear synaptopathy: An early-onset contributor to auditory functional decline. J. Neurosci..

[6] Möhrle D., Ni K., Varakina K., Bing D., Lee S.C., Zimmermann U., Knipper M., Rüttiger L. (2016). Loss of auditory sensitivity from inner hair cell synaptopathy can be centrally compensated in the young but not old brain. Neurobiol. Aging.

[7] Viana L.M., O’Malley J.T., Burgess B.J., Jones D.D., Oliveira C.A., Santos F., Merchant S.N., Liberman L.D., Liberman M.C. (2015). Cochlear neuropathy in human presbycusis: Confocal analysis of hidden hearing loss in post-mortem tissue. Hear. Res..

[8] Liberman M.C., Kujawa S.G. (2017). Cochlear synaptopathy in acquired sensorineural hearing loss: Manifestations and mechanisms. Hear. Res..

[9] Kobel M., Le Prell C.G., Liu J., Hawks J.W., Bao J. (2017). Noise-induced cochlear synaptopathy: Past findings and future studies. Hear. Res..

[10] Furman A.C., Kujawa S.G., Liberman M.C. (2013). Noise-induced cochlear neuropathy is selective for fibers with low spontaneous rates. J. Neurophysiol..

[11] Schmiedt R.A., Mills J.H., Boettcher F.A. (1996). Age-related loss of activity of auditory-nerve fibers. J. Neurophysiol..

[12] Parthasarathy A., Kujawa S.G. (2018). Synaptopathy in the aging cochlea: Characterizing early-neural deficits in auditory temporal envelope processing. J. Neurosci..

[13] Ridley C.L., Kopun J.G., Neely S.T., Gorga M.P., Rasetshwane D.M. (2018). Using thresholds in noise to identify hidden hearing loss in humans. Ear Hear..

[14] Heinz M.G., Young E.D. (2004). Response growth with sound level in auditory-nerve fibers after noise-induced hearing loss. J. Neurophysiol..

[15] Heinz M.G., Issa J.B., Young E.D. (2005). Auditory-nerve rate responses are inconsistent with common hypotheses for the neural correlates of loudness recruitment. J. Assoc. Res. Otolaryngol..

[16] Ruel J., Chabbert C., Nouvian R., Bendris R., Eybalin M., Leger C.L., Bourien J., Mersel M., Puel J.L. (2008). Salicylate enables cochlear arachidonic-acid-sensitive NMDA receptor responses. J. Neurosci..

[17] King K., Stephens D. (1992). Auditory and psychological factors in ‘auditory disability with normal hearing’. Scand. Audiol..

[18] Saunders G.H., Field D.L., Haggard M.P. (1992). A clinical test battery for obscure auditory dysfunction (OAD): Development, selection and use of tests. Br. J. Audiol..

[19] Stephens D., Zhao F. (2000). The role of a family history in king kopetzky syndrome (obscure auditory dysfunction). Acta Otolaryngol..

[20] Kuwada S., Anderson J.S., Batra R., Fitzpatrick D.C., Teissier N., D’Angelo W.R. (2002). Sources of the scalp-recorded amplitude-modulation following response. J. Am. Acad. Audiol..

[21] Bidet-Caulet A., Fischer C., Besle J., Aguera P.E., Giard M.H., Bertrand O. (2007). Effects of selective attention on the electrophysiological representation of concurrent sounds in the human auditory cortex. J. Neurosci..

[22] Brugge J.F., Nourski K.V., Oya H., Reale R.A., Kawasaki H., Steinschneider M., Howard M.A. (2009). Coding of repetitive transients by auditory cortex on Heschl’s gyrus. J. Neurophysiol..

[23] Grose J.H., Buss E., Elmore H. (2019). Age-related changes in the auditory brainstem response and suprathreshold processing of temporal and spectral modulation. Trends Hear..

[24] Matt L., Eckert P., Panford-Walsh R., Geisler H.S., Bausch A.E., Manthey M., Muller N.I.C., Harasztosi C., Rohbock K., Ruth P. (2018). Visualizing BDNF transcript usage during sound-induced memory linked plasticity. Front. Mol. Neurosci..

[25] Kamerer A.M., AuBuchon A., Fultz S.E., Kopun J.G., Neely S.T., Rasetshwane D.M. (2019). The role of cognition in common measures of peripheral synaptopathy and hidden hearing loss. Am. J. Audiol..

[26] Singer W., Manthey M., Panford-Walsh R., Matt L., Geisler H.S., Passeri E., Baj G., Tongiorgi E., Leal G., Duarte C.B. (2018). BDNF-live-exon-visualization (BLEV) allows differential detection of BDNF transcripts in vitro and in vivo. Front. Mol. Neurosci..

[27] Melcher J.R., Kiang N.Y. (1996). Generators of the brainstem auditory evoked potential in cat. III: Identified cell populations. Hear. Res..

[28] Engel J., Braig C., Rüttiger L., Kuhn S., Zimmermann U., Blin N., Sausbier M., Kalbacher H., Münkner S., Rohbock K. (2006). Two classes of outer hair cells along the tonotopic axis of the cochlea. Neuroscience.

[29] Burkard R.F., Don M., Burkard R.F., Eggermont J.J., Don M. (2007). The auditory brainstem response. Auditory Evoked Potentials: Basic Principles and Clinical Application.

[30] Matt L., Michalakis S., Hofmann F., Hammelmann V., Ludwig A., Biel M., Kleppisch T. (2011). HCN2 channels in local inhibitory interneurons constrain LTP in the hippocampal direct perforant path. Cell. Mol. Life Sci..

[31] Chenaux G., Matt L., Hill T.C., Kaur I., Liu X.-B., Kirk L.M., Speca D.J., McMahon S.A., Zito K., Hell J.W. (2016). Loss of SynDIG1 reduces excitatory synapse maturation but not formation in vivo. SynDIG1 regulates excitatory synapse maturation. ENeuro.

[32] Singer W., Geisler H.S., Panford-Walsh R., Knipper M. (2016). Detection of excitatory and inhibitory synapses in the auditory system using fluorescence immunohistochemistry and high-resolution fluorescence microscopy. Methods Mol. Biol..

[33] Zampini V., Johnson S.L., Franz C., Lawrence N.D., Munkner S., Engel J., Knipper M., Magistretti J., Masetto S., Marcotti W. (2010). Elementary properties of CaV1.3 Ca(2+) channels expressed in mouse cochlear inner hair cells. J. Physiol..

[34] Chumak T., Rüttiger L., Lee S.C., Campanelli D., Zuccotti A., Singer W., Popelar J., Gutsche K., Geisler H.S., Schraven S.P. (2016). BDNF in lower brain parts modifies auditory fiber activity to gain fidelity but increases the risk for generation of central noise after injury. Mol. Neurobiol..

[35] Marchetta P., Mohrle D., Eckert P., Reimann K., Wolter S., Tolone A., Lang I., Wolters M., Feil R., Engel J. (2020). Guanylyl cyclase A/cGMP signaling slows hidden, age- and acoustic trauma-induced hearing loss. Front. Aging Neurosci..

[36] Gleich O., Semmler P., Strutz J. (2016). Behavioral auditory thresholds and loss of ribbon synapses at inner hair cells in aged gerbils. Exp. Gerontol..

[37] Valero M.D., Burton J.A., Hauser S.N., Hackett T.A., Ramachandran R., Liberman M.C. (2017). Noise-induced cochlear synaptopathy in rhesus monkeys (Macaca mulatta). Hear. Res..

[38] Wu P.Z., Liberman L.D., Bennett K., de Gruttola V., O’Malley J.T., Liberman M.C. (2019). Primary neural degeneration in the human cochlea: Evidence for hidden hearing loss in the aging ear. Neuroscience.

[39] Johnson D.H., Kiang N.Y. (1976). Analysis of discharges recorded simultaneously from pairs of auditory nerve fibers. Biophys. J..

[40] Buran B.N., Strenzke N., Neef A., Gundelfinger E.D., Moser T., Liberman M.C. (2010). Onset coding is degraded in auditory nerve fibers from mutant mice lacking synaptic ribbons. J. Neurosci..

[41] Jaumann M., Dettling J., Gubelt M., Zimmermann U., Gerling A., Paquet-Durand F., Feil S., Wolpert S., Franz C., Varakina K. (2012). cGMP-Prkg1 signaling and Pde5 inhibition shelter cochlear hair cells and hearing function. Nat. Med..

[42] Rüttiger L., Zimmermann U., Knipper M. (2017). Biomarkers for hearing dysfunction: facts and outlook. ORL.

[43] Khimich D., Nouvian R., Pujol R., Tom Dieck S., Egner A., Gundelfinger E.D., Moser T. (2005). Hair cell synaptic ribbons are essential for synchronous auditory signalling. Nature.

[44] Rhode W.S., Smith P.H. (1986). Encoding timing and intensity in the ventral cochlear nucleus of the cat. J. Neurophysiol..

[45] Rhode W.S., Smith P.H. (1986). Physiological studies on neurons in the dorsal cochlear nucleus of cat. J. Neurophysiol..

[46] Hu H., Gan J., Jonas P. (2014). Interneurons. Fast-spiking, parvalbumin^+^ GABAergic interneurons: From cellular design to microcircuit function. Science.

[47] Ouda L., Profant O., Syka J. (2015). Age-related changes in the central auditory system. Cell Tissue Res..

[48] Williamson T.T., Zhu X., Walton J.P., Frisina R.D. (2015). Auditory brainstem gap responses start to decline in mice in middle age: A novel physiological biomarker for age-related hearing loss. Cell Tissue Res..

[49] Frisina D.R., Frisina R.D. (1997). Speech recognition in noise and presbycusis: Relations to possible neural mechanisms. Hear. Res..

[50] Kujawa S.G., Liberman M.C. (2015). Synaptopathy in the noise-exposed and aging cochlea: Primary neural degeneration in acquired sensorineural hearing loss. Hear. Res..

[51] Bourien J., Tang Y., Batrel C., Huet A., Lenoir M., Ladrech S., Desmadryl G., Nouvian R., Puel J.L., Wang J. (2014). Contribution of auditory nerve fibers to compound action potential of the auditory nerve. J. Neurophysiol..

[52] Frisina R.D., Karcich K.J., Tracy T.C., Sullivan D.M., Walton J.P., Colombo J. (1996). Preservation of amplitude modulation coding in the presence of background noise by chinchilla auditory-nerve fibers. J. Acoust. Soc. Am..

[53] Grose J.H., Buss E., Hall J.W. (2017). Loud music exposure and cochlear synaptopathy in young adults: Isolated auditory brainstem response effects but no perceptual consequences. Trends Hear..

[54] Prendergast G., Guest H., Munro K.J., Kluk K., Leger A., Hall D.A., Heinz M.G., Plack C.J. (2017). Effects of noise exposure on young adults with normal audiograms I: Electrophysiology. Hear. Res..

[55] Yeend I., Beach E.F., Sharma M., Dillon H. (2017). The effects of noise exposure and musical training on suprathreshold auditory processing and speech perception in noise. Hear. Res..

[56] Yates G.K. (1991). Auditory-nerve spontaneous rates vary predictably with threshold. Hear. Res..

[57] Knipper M., van Dijk P., Schulze H., Mazurek B., Krauss P., Scheper V., Warnecke A., Schlee W., Schwabe k., Singer W. (2020). The neural bases of tinnitus: Lessons from deafness and cochlear implants. J. Neurosci..

[58] Schaette R., Kempter R. (2009). Predicting tinnitus pitch from patients’ audiograms with a computational model for the development of neuronal hyperactivity. J. Neurophysiol..

[59] Schaette R., Kempter R. (2012). Computational models of neurophysiological correlates of tinnitus. Front. Syst. Neurosci..

[60] Caspary D.M., Milbrandt J.C., Helfert R.H. (1995). Central auditory aging: GABA changes in the inferior colliculus. Exp. Gerontol..

[61] Levakova M., Tamborrino M., Ditlevsen S., Lansky P. (2015). A review of the methods for neuronal response latency estimation. Biosystems.

[62] Heeringa A.N., van Dijk P. (2014). The dissimilar time course of temporary threshold shifts and reduction of inhibition in the inferior colliculus following intense sound exposure. Hear. Res..

[63] Cai S., Ma W.L., Young E.D. (2009). Encoding intensity in ventral cochlear nucleus following acoustic trauma: Implications for loudness recruitment. J. Assoc. Res. Otolaryngol..

[64] Irvine D.R.F. (2018). Plasticity in the auditory system. Hear. Res..

[65] Kraus N., White-Schwoch T. (2015). Unraveling the biology of auditory learning: A cognitive-sensorimotor-reward framework. Trends Cogn. Sci..

[66] Kilgard M.P., Pandya P.K., Engineer N.D., Moucha R. (2002). Cortical network reorganization guided by sensory input features. Biol. Cybern..

[67] Schonwiesner M., Novitski N., Pakarinen S., Carlson S., Tervaniemi M., Naatanen R. (2007). Heschl’s gyrus, posterior superior temporal gyrus, and mid-ventrolateral prefrontal cortex have different roles in the detection of acoustic changes. J. Neurophysiol..

[68] Malmierca M.S., Sanchez-Vives M.V., Escera C., Bendixen A. (2014). Neuronal adaptation, novelty detection and regularity encoding in audition. Front. Syst. Neurosci..

[69] Weinberger N.M. (2015). New perspectives on the auditory cortex: Learning and memory. Handb. Clin. Neurol..

[70] de Kloet E.R. (2014). From receptor balance to rational glucocorticoid therapy. Endocrinology.

[71] Irvine D.R.F. (2018). Auditory perceptual learning and changes in the conceptualization of auditory cortex. Hear. Res..

[72] Viho E.M.G., Buurstede J.C., Mahfouz A., Koorneef L.L., van Weert L., Houtman R., Hunt H.J., Kroon J., Meijer O.C. (2019). Corticosteroid action in the brain: The potential of selective receptor modulation. Neuroendocrinology.

[73] Kaltenbach J.A., Zhang J. (2007). Intense sound-induced plasticity in the dorsal cochlear nucleus of rats: Evidence for cholinergic receptor upregulation. Hear. Res..

[74] Salvi R.J., Wang J., Ding D. (2000). Auditory plasticity and hyperactivity following cochlear damage. Hear. Res..

[75] Wang H., Brozoski T.J., Turner J.G., Ling L., Parrish J.L., Hughes L.F., Caspary D.M. (2009). Plasticity at glycinergic synapses in dorsal cochlear nucleus of rats with behavioral evidence of tinnitus. Neuroscience.

[76] Dehmel S., Pradhan S., Koehler S., Bledsoe S., Shore S. (2012). Noise overexposure alters long-term somatosensory-auditory processing in the dorsal cochlear nucleus—Possible basis for tinnitus-related hyperactivity?. J. Neurosci..

[77] Groschel M., Ryll J., Gotze R., Ernst A., Basta D. (2014). Acute and long-term effects of noise exposure on the neuronal spontaneous activity in cochlear nucleus and inferior colliculus brain slices. Biomed. Res. Int..

[78] Vogler D.P., Robertson D., Mulders W.H. (2011). Hyperactivity in the ventral cochlear nucleus after cochlear trauma. J. Neurosci..

[79] Sowell E.R., Delis D., Stiles J., Jernigan T.L. (2001). Improved memory functioning and frontal lobe maturation between childhood and adolescence: A structural MRI study. J. Int. Neuropsychol. Soc..

[80] Nunez A., Malmierca E. (2007). Corticofugal modulation of sensory information. Adv. Anat. Embryol. Cell Biol..

[81] Wittekindt A., Kaiser J., Abel C. (2014). Attentional modulation of the inner ear: A combined otoacoustic emission and EEG study. J. Neurosci..

[82] Dragicevic C.D., Marcenaro B., Navarrete M., Robles L., Delano P.H. (2019). Oscillatory infrasonic modulation of the cochlear amplifier by selective attention. PLoS ONE.

[83] Miller E.K., Buschman T.J. (2013). Cortical circuits for the control of attention. Curr. Opin. Neurobiol..

[84] Singer W., Gröschel M., Zuccotti A., Mueller S., Ernst A., Basta D., Knipper M., Rüttiger L. (2020). The aftermath of tinnitus-inducing inner ear damage for auditory brainstem responses and MEMR imaging of central brain activity in the rat. Hear. Balance Commun. Artic..

[85] Arango-Lievano M., Borie A.M., Dromard Y., Murat M., Desarmenien M.G., Garabedian M.J., Jeanneteau F. (2019). Persistence of learning-induced synapses depends on neurotrophic priming of glucocorticoid receptors. Proc. Natl. Acad. Sci. USA.

[86] Dieni S., Matsumoto T., Dekkers M., Rauskolb S., Ionescu M.S., Deogracias R., Gundelfinger E.D., Kojima M., Nestel S., Frotscher M. (2012). BDNF and its pro-peptide are stored in presynaptic dense core vesicles in brain neurons. J. Cell Biol..

[87] Chacon-Fernandez P., Sauberli K., Colzani M., Moreau T., Ghevaert C., Barde Y.A. (2016). Brain-derived neurotrophic factor in megakaryocytes. J. Biol. Chem..

[88] Hillman E.M. (2014). Coupling mechanism and significance of the BOLD signal: A status report. Annu. Rev. Neurosci..

[89] Broadway J.M., Engle R.W. (2011). Lapsed attention to elapsed time? Individual differences in working memory capacity and temporal reproduction. Acta Psychol..

[90] Fullgrabe C., Moore B.C., Stone M.A. (2014). Age-group differences in speech identification despite matched audiometrically normal hearing: Contributions from auditory temporal processing and cognition. Front. Aging Neurosci..

[91] Tucsek Z., Valcarcel-Ares M.N., Tarantini S., Yabluchanskiy A., Fulop G., Gautam T., Orock A., Csiszar A., Deak F., Ungvari Z. (2017). Hypertension-induced synapse loss and impairment in synaptic plasticity in the mouse hippocampus mimics the aging phenotype: Implications for the pathogenesis of vascular cognitive impairment. Geroscience.

[92] Shi Y., Thrippleton M.J., Makin S.D., Marshall I., Geerlings M.I., de Craen A.J.M., van Buchem M.A., Wardlaw J.M. (2016). Cerebral blood flow in small vessel disease: A systematic review and meta-analysis. J. Cereb. Blood Flow Metab..

[93] Lin V.Y., Chung J., Callahan B.L., Smith L., Gritters N., Chen J.M., Black S.E., Masellis M. (2017). Development of cognitive screening test for the severely hearing impaired: Hearing-impaired MoCA. Laryngoscope.

[94] Livingston G., Frankish H. (2015). A global perspective on dementia care: A lancet commission. Lancet.

[95] Montero-Odasso M., Ismail Z., Livingston G. (2020). One third of dementia cases can be prevented within the next 25 years by tackling risk factors. The case “for” and “against”. Alzheimer’s Res. Ther..

