# Supplementary material for: Age-Dependent Auditory Processing Deficits after Cochlear Synaptopathy Depend on Auditory Nerve Latency and the Ability of the Brain to Recruit LTP/BDNF
Source: Brain Sci. 2020 Oct 6;10(10):710. doi: 10.3390/brainsci10100710 (PMC7601375; doi:10.3390/brainsci10100710)
Supplement: Supplementary file 1 [file brainsci-10-00710-s001.pdf]

Supplementary Materials: Age-Dependent Auditory Processing Deficits after Cochlear Synaptopathy Depend on Auditory Nerve Latency and the Ability of the Brain to Recruit LTP/BDNF

Philine Marchetta, Daria Savitska, Angelika Kübler, Giulia Asola, Marie Manthey, Dorit Möhrle, Thomas Schimmang, Lukas Rüttiger, Marlies Knipper and Wibke Singer

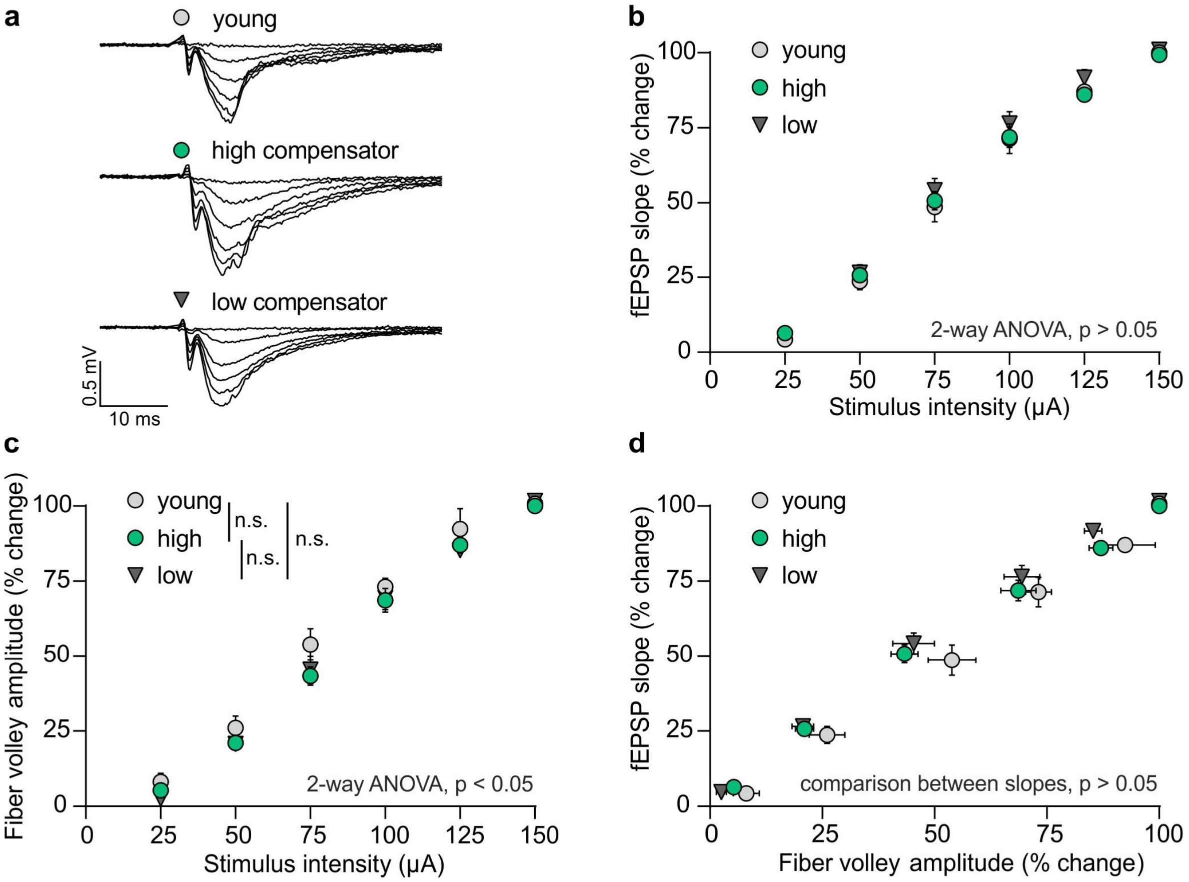

Supplementary Figure S1. Input-output relationship between fEPSP slope, fiber volley amplitude and stimulus intensity. (**a**) Representative traces of fEPSPs with increasing stimulus intensity (from 25 µA to 150 µA in 25 µA steps). (**b**) Changes in fEPSP slope and (**c**) fiber volley amplitude were normalized within each slice (% from the maximal response at the highest stimulus strength) and averaged values for each group were plotted against the stimulus intensity. Both, changes of fEPSPs slopes (2-way ANOVA, $F(2, 300) = 1.446$, $p > 0.05$) and fiber volley amplitudes (2-way ANOVA, $F(2, 300) = 4.127$, $p < 0.05$; with two-stage linear step-up procedure of Benjamini, Krieger and Yekutieli: high compensator vs. low compensator, $p > 0.05$; young vs. high compensator, $p > 0.05$; young vs. low compensator, $p > 0.05$) showed no differences between the low and highly compensating animals as well as young control animals. (**d**) Changes in fEPSP slopes were consistent with changes in fiber volley amplitudes within all groups, displaying stable synaptic transmission (simple linear regression analysis, comparison between slopes of lines, $F(2, 323) = 0.69$, $p > 0.05$). Mean ± SEM. n animals/slices: young 7/21, high compensator 7/20, low compensator 5/12.

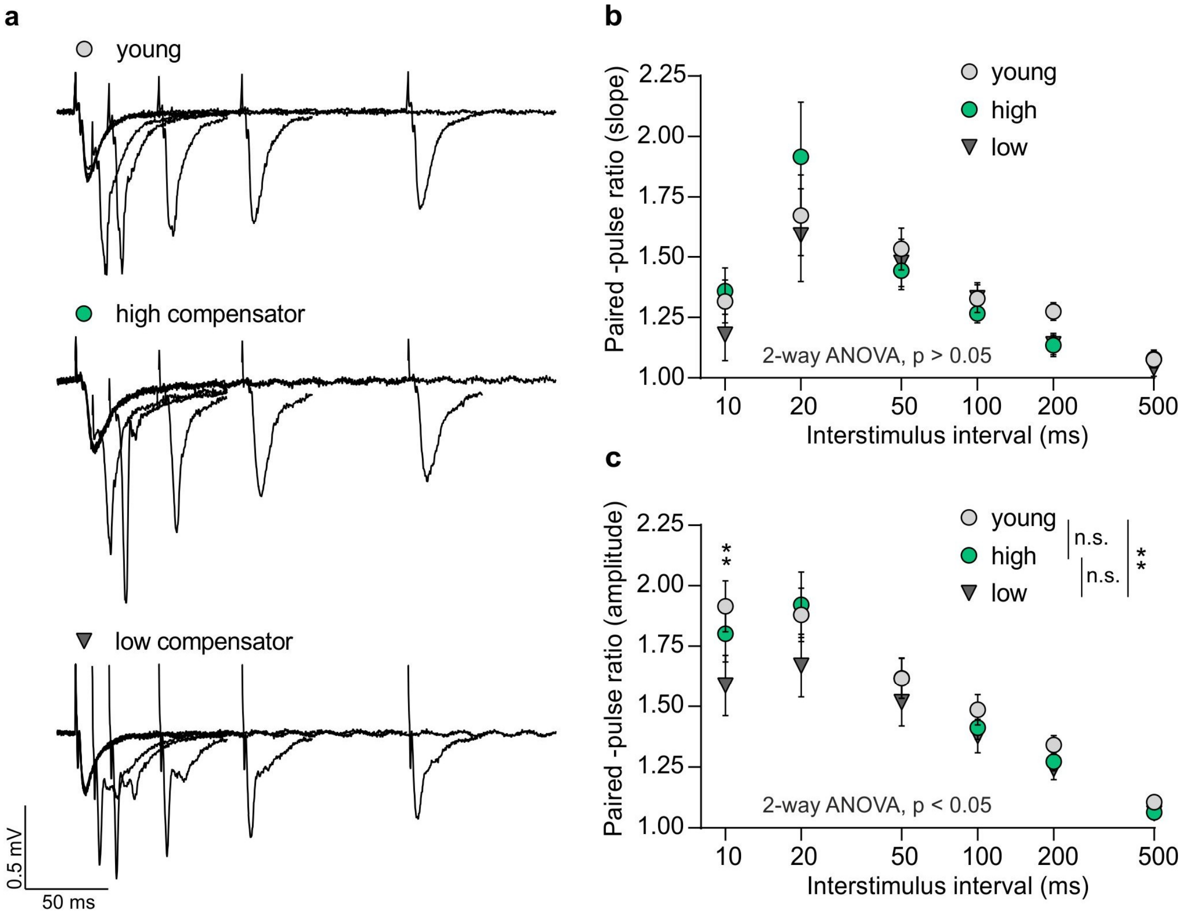

Supplementary Figure S2. Paired-pulse facilitation (PPF) as an indicator of short-term plasticity. (**a**) Representative traces of fEPSPs with increasing interpulse interval at the stimulation strength equal to respective LTP recordings. Applied interstimulus intervals: 10 ms, 20 ms, 50 ms, 100 ms, 200 ms, 500 ms (not shown). (**b**) Paired-pulse ratio of EPSP2/EPSP1 slope (2-way ANOVA, F (2, 330) = 0.9445, $p > 0.05$) and (**c**) paired-pulse ratio of EPSP2/EPSP1 amplitude (2-way ANOVA, F (2, 330) = 4.487, $p < 0.05$; with two-stage linear step-up procedure of Benjamini, Krieger and Yekutieli: high compensator vs. low compensator, $p > 0.05$) showed no prominent difference between highly compensating and low compensating animals at any applied interstimulus interval, indicating similar short-term potentiation between these groups. Paired-pulse ratio of EPSP2/EPSP1 amplitude was significantly different between young (1.88 ± 0.11; n = 7/21 animals/slices) and low compensating (1.66 ± 0.14; n = 5/16 animals/slices) animals only at the 10 ms interpulse interval. Otherwise paired-pulse facilitation was similar in young mice compared to highly or low compensating animals (2-way ANOVA, F (2, 330) = 4.487, $p < 0.05$; with two-stage linear step-up procedure of Benjamini, Krieger and Yekutieli: young vs. high compensator, $p > 0.05$; young vs. low compensator, $p < 0.01$ only at 10 ms interpulse interval; otherwise, young vs. low compensator, $p > 0.05$). EPSP1 was calculated as an average of EPSP1s from all interstimulus intervals for each single slice. Mean ± SEM. n animals/slices: young 7/21, high compensator 7/20, low compensator 5/16.
